# Supplementary material for: Glycoproteomic and proteomic analysis of Burkholderia cenocepacia reveals glycosylation events within FliF and MotB are dispensable for motility
Source: Microbiol Spectr. 2024 May 6;12(6):e00346-24. doi: 10.1128/spectrum.00346-24 (PMC11237607; doi:10.1128/spectrum.00346-24)
Supplement: Supplemental material — Tables S10 to S13; Fig. S1 to S24. [file spectrum.00346-24-s0001.pdf]

Glycoproteomic and Proteomic analysis of *Burkholderia cenocepacia* reveals glycosylation events within FliF and MotB are dispensable for motility.

Jessica M. Lewis[1,2*], Leila Jebeli[1*], Pauline M.L. Coulon[1,3*], Catrina E. Lay[1], and Nichollas E. Scott[1#]

[1]Department of Microbiology and Immunology, University of Melbourne at the Peter Doherty Institute for Infection and Immunity, Melbourne 3000, Australia

Current addresses:

[2]School of Life Sciences, University of Warwick, Coventry, UK.

[3]Australian Institute for Microbiology and Immunology, Faculty of Science, University of Technology Sydney, Ultimo 2007, Australia

[#]To whom correspondence and requests for materials should be addressed N.E.S (Nichollas.scott@unimelb.edu.au)

* These authors contributed equally to this publication

Key words: Glycosylation, *Burkholderia cenocepacia*, *Burkholderia*, Post-translational modifications, Proteomics, PglL, Glycoproteomics

Table of Contents

Title	Page
**Supplementary Table Legends 1-9**	**4**
**Supplementary Table 10: Strain list**	**7**
**Supplementary Table 11: Plasmid list**	**8**
**Supplementary Table 12: Primer list**	**9**
**Supplementary Table 13: Proteomic datasets**	**11**
**Supplementary Figure 1. Growth curves and CFU per ml counts of strains across the different media used within this study**	**13**
**Supplementary Figure 2. Comparison of ZIC-HILIC and FAIMS glycopeptide enrichment approaches**	**14**
**Supplementary Figure 3. Unique glycoproteins identified within this study across different growth media**	**15**
**Supplementary Figure 4. *B. cenocepacia* glycoproteome GO term and functional assignments**	**16**
**Supplementary Figure 5. Pearson correlation and PCA analysis of *B. cenocepacia* proteomes across growth phases within different media**	**17**
**Supplementary Figure 6. Precursor coverage within *B. cenocepacia* proteomes across growth phases and different media**	**18**
**Supplementary Figure 7. Observed fold changes within the *B. cenocepacia* Proteome and glycoproteome across growth phases and different media**	**19**
**Supplementary Figure 8. Protein levels of observed glycosylation machinery across growth phases and different media**	**20**
**Supplementary Figure 9. Pearson correlation and PCA analysis of *B. cenocepacia* proteomes differing in the presence of glycosylation across growth phases**	**21**
**Supplementary Figure 10. Coverage of the *B. cenocepacia* proteome observed across different media and coverage of the known glycoproteome**	**22**
**Supplementary Figure 11. Proteome changes observed within Δ*pglL* compared to WT under different growth phases**	**23**
**Supplementary Figure 12. Analysis of *B. cenocepacia* glycoproteins observed to increase in abundance using DIA analysis**	**24**
**Supplementary Figure 13. Confirmation of *B. cenocepacia* Δ*motB* strains using DDA analysis.**	**25**
**Supplementary Figure 14. Confirmation of *B. cenocepacia* Δ*fliF* strains using DDA analysis.**	**26**
**Supplementary Figure 15. Confirmation of *B. cenocepacia* ΔBCAM0505 strains using DDA analysis**	**27**
**Supplementary Figure 16. Protein levels of MotB, FliF,, and BCAM0505 across strains.**	**28**

**Supplementary Figure 17. QC plots of Δ*motB*, Δ*fliF*, Δ*BCAM0505,* WT and Δ*pglL* proteomes assessed using DIA analysis**	**29**
**Supplementary Figure 18. Overlap in proteins altered observed in Δ*motB*, Δ*fliF*, ΔBCAM0505 verses WT compared to Δ*pglL* verses WT**	**30**
**Supplementary Figure 19. Enrichment analysis of GO terms associated with altered proteins in Δ*motB*, Δ*fliF*, Δ*BCAM0505*, Δ*pglL* verses WT.**	**31**
**Supplementary Figure 20. Overexpression of *motB* from pSCrhaB2 leads to motility defects in *B. cenocepacia* K56-2**	**32**
**Supplementary Figure 21. QC plots of DIA analysis of *B. cenocepacia* strains containing FliF complementation vectors and controls.**	**33**
**Supplementary Figure 22. QC plots of DIA analysis of *B. cenocepacia* strains containing MotB complementation vectors and controls.**	**34**
**Supplementary Figure 23. Overexpression of *motB* or fliF from pSCrhaB2 does not restore motility in *B. cenocepacia* K56-2 Δ*pglL***	**35**
**Supplementary Figure 24. Uncropped Western blotting images from Figure 6C and D.**	**36**
**References**	**37**

Supplementary tables

Supplementary Table 1. Glycopeptide identifications within *B. cenocepacia* K56-2 identified across growth conditions using FAIMS and ZIC-HILIC enrichments. Combined MSFragger PSM peptide level search results of all potential glycopeptides identified across biological replicates (n=4) and growth conditions using FAIMS and ZIC-HILIC enrichment. For all identified glycopeptides the spectrum, data files, peptide sequence, peptide length, charge, retention times, observed m/z, calibrated m/z, observed mass, calibrated mass, delta mass, scores, peptide position within protein, assigned modifications, glycan assignment quality, protein, protein.ID, Culturing condition(media), replicate number and compensation voltage (for glycopeptides identified using FAIMS) are provided. The lists of unique glycopeptides observed across replicates as well as the manually curated glycopeptides spectra used for the creation of Supplementary Data 1 are provided [DOI: 10.6084/m9.figshare.25492774].

Supplementary Table 2. Glycoproteins identified across *B. cenocepacia.* Curated list of all known glycoproteins within *B. cenocepacia* including confirmed glycosylation sites, residue of the assigned glycosylation site and if the glycoproteins have been previously identified within previous studies. For each glycoprotein the location and number of predicted transmembrane domains, location of the Signal tag and GO terms associated with each protein are provided derived from the *B. cenocepacia* J2315 proteome (UP000001035). Byonic assigned glycopeptides identified using FAIMS and ZIC-HILIC enrichment from prior studies of J2315 [39,43] not previously manually validated have been curated to ensure only confirmed glycoproteins are considered in our DIA analysis. For these glycopeptides the Byonic assigned spectra in addition to peptide assignment associated metrics (m/z; observed m/z, score, position in protein, Byonic score), *B. cenocepacia* J2315 gene assignment and if the glycosylation site was localised are provided. The lists of unique glycopeptides observed within these studies and the unique manually curated glycopeptides spectra identified from these studies within Supplementary Data 2 are provided [DOI: 10.6084/m9.figshare.25492774].

Supplementary Table 3. DIA Protein level analysis of *B. cenocepacia* K56-2 grown in LB, TSB and ASM culturing media (Stationary and Log growth phases). The Spectronaut search results for protein level analysis of biological replicates (n=4) grown in LB, TSB and ASM at log and stationary phase. Both the raw Spectronaut outputs and Perseus imputed data with statistical analysis processed data are provided. For each identified protein, the $\log_2$ LFQ protein values, T-test information including the $-\log_{10}$(*p*-value), difference in the mean between the groups and if the resulting *p*-values are below 0.05, the multiple hypothesis corrected *p*-values (permutation-based false discovery rate of 0.05) are provided. Categorical information associated with protein accessions, gene name, and GO terms are provided in addition to if proteins were identified by a single PSM within a given experiment as well as the total number of precursors assigned for each protein within each replicate.

Supplementary Table 4. DIA Protein level analysis of *B. cenocepacia* K56-2 wildtype, Δ*pglL and* Δ*pglL*Δ*amrAB::native-promotor-pglL-his* grown to stationary phase in LB, TSB and ASM culturing media. The Spectronaut search results for protein level analysis of biological replicates (n=4) grown in LB, TSB and ASM to stationary phase. Both the raw Spectronaut outputs and

Perseus imputed data with statistical analysis processed data are provided. For each identified protein, the $\log_2$ LFQ protein values, T-test information including the -$\log_{10}$(*p*-value), difference in the mean between the groups and if the resulting *p*-values are below 0.05, the multiple hypothesis corrected *p*-values (permutation-based false discovery rate of 0.05) are provided. Categorical information associated with protein accessions, gene name, and GO terms are provided in addition to if proteins were identified by a single PSM within a given experiment as well as the total number of precursors assigned for each protein within each replicate.

Supplementary Table 5. DDA protein level LFQ analysis of *B. cenocepacia* Δ*motB* candidates; Δ*fliF* candidates and Δ*BCAM0505* candidates compared to WT at stationary phase in LB. The Perseus processed MaxQuant search results for the protein analysis of four biological replicates of strains WT, mutant candidate A and mutant candidate B are provided. For each identified protein, the log2 LFQ protein values, the T-test information including the -$\log_{10}$(*p*-value), difference in the mean between the groups and if the resulting *p*-values are below the multiple hypothesis corrected *p*-value are provided. For each protein the protein score, number of MS/MS events for the corresponding protein, expected molecular weight, number of peptides identified, peptide sequence coverage and if a given protein was identified within the biological replicate by MS/MS data or by matching between runs are provided. For each set of mutants both the imputed and non-imputed data has been provided.

Supplementary Table 6. DIA Protein level analysis of Δ*motB,* Δ*fliF,* ΔBCAM0505 *and* Δ*pglL* compared to WT at stationary phase in LB. The Spectronaut search results for protein level analysis of biological replicates (n=4) grown in LB at stationary phase for *motB,* Δ*fliF,* ΔBCAM0505*,* Δ*pglL* and WT. Both the raw Spectronaut outputs and Perseus imputed data with statistical analysis are provided. For each identified protein, the $\log_2$ LFQ protein values, T-test information including the -$\log_{10}$(*p*-value), difference in the mean between the groups and if the resulting *p*-values are below 0.05, the multiple hypothesis corrected *p*-values (permutation-based false discovery rate of 0.05) are provided. Categorical information associated with protein accessions, gene name, and GO terms are provided in addition to if proteins were identified by a single PSM within a given experiment as well as the total number of precursors assigned for each protein within each replicate.

Supplementary Table 7. Enrichment analysis of proteome changes observed within Δ*motB,* Δ*fliF* and Δ*pglL* compared to WT at stationary phase in LB*.* Fisher exact tests outputs assessing the co-occurrence of categorical assignments (statistically significant changes to proteins observed within strains or GO terms) between different strains.

Supplementary Table 8. DIA Protein level analysis of Δ*motB* and WT containing pScrhaB2, pSCrhaB2-*motB* or pSCrhaB2-*motB*$_{S331A}$ with / without induction (0.1% rhamnose) at stationary phase in LB. The Spectronaut search results for protein level analysis of biological replicates (n=4) grown in LB at stationary phase +/- the addition of Rhamnose of Δ*motB and WT* containing either pScrhaB2*,* pSCrhaB2-*motB* or pSCrhaB2-*motB*$_{S331A}$. Both the raw Spectronaut outputs and Perseus imputed data with statistical analysis are provided. For each identified protein, the $\log_2$

LFQ protein values, T-test information including the -log_{10}(*p*-value), difference in the mean between the groups and if the resulting *p*-values are below 0.05, the multiple hypothesis corrected *p*-values (permutation-based false discovery rate of 0.05) are provided. Categorical information associated with protein accessions, gene name, and GO terms are provided in addition to if proteins were identified by a single PSM within a given experiment as well as the total number of precursors assigned for each protein within each replicate.

Supplementary Table 9. DIA Protein level analysis of Δ*fliF* containing pScrhaB2, pSCrhaB2-*fliF* or pSCrhaB2-*fliF*$_{S358A}$ and WT containing pScrhaB2 with / without induction (0.1% rhamnose) at stationary phase in LB. The Spectronaut search results for protein level analysis of biological replicates (n=4) grown in LB at stationary phase +/- the addition of rhamnose of Δ*fliF* containing either pScrhaB2*,* pSCrhaB2-*fliF* or pSCrhaB2-*fliF*$_{S358A}$ and WT containing pScrhaB2. Both the raw Spectronaut outputs and Perseus imputed data with statistical analysis are provided. For each identified protein, the log_2 LFQ protein values, T-test information including the -log_{10}(*p*-value), difference in the mean between the groups and if the resulting *p*-values are below 0.05, the multiple hypothesis corrected *p*-values (permutation-based false discovery rate of 0.05) are provided. Categorical information associated with protein accessions, gene name, and GO terms are provided in addition to if proteins were identified by a single PSM within a given experiment as well as the total number of precursors assigned for each protein within each replicate.

Supplementary Table 10: Strain list

Strain name	Description	Source/ Reference
***E. coli* strain**		
E. coli pir2	F^{-} Δ*lac169 rpoS*(*Am*) *robA1 creC510 hsdR514 endA recA1 uidA*(Δ*MluI*)::*pir-116*	Thermo Scientific
***Burkholderia* strains**		
B. cenocepacia K56-2	Clinical isolate of the ET12 lineage (Darling P 1998, Mahenthiralingam E *et al* 2005)	Canadian *B. cepacia* research and referral repository [1]
B. cenocepacia K56-2 Δ*pglL*	Δ*pglL* (BCAL0960) derivative of K56-2 created using pYM8	[2]
B. cenocepacia K56-2 Δ*pglL amrAB::native-pglL-his*$_{10}$	Δ*amrAB::native-pglL-his*$_{10}$ chromosomal complement derivative of Δ*pglL* (BCAL0960) expressing *pglL* from the native promoter	[2]
B. cenocepacia K56-2 Δ*fliF* A	Δ*fliF* (BCAL0525) derivative of K56-2 created using pGPI-SceI-BCAL0525. Independent mutant A	This study
B. cenocepacia K56-2 Δ*fliF* B	Δ*fliF* (BCAL0525) derivative of K56-2 created using pGPI-SceI-BCAL0525. Independent mutant B	This study
B. cenocepacia K56-2 ΔBCAM0505 A	ΔBCAM0505 derivative of K56-2 created using pGPI-SceI-BCAM0505. Independent mutant A	This study
B. cenocepacia K56-2 ΔBCAM0505 B	ΔBCAM0505 derivative of K56-2 created using pGPI-SceI-BCAM0505. Independent mutant B	This study
B. cenocepacia K56-2 Δ*motB* A	Δ*motB* (BCAL0127) derivative of K56-2 created using pGPI-SceI-BCAL0127. Independent mutant A	This study
B. cenocepacia K56-2 Δ*motB* B	Δ*motB* (BCAL0127) derivative of K56-2 created using pGPI-SceI-BCAL0127. Independent mutant B	This study
B. cenocepacia K56-2 Δ*pglL*Δ*fliF*	Δ*pglL*Δ*fliF* derivative of K56-2 created from *B. cenocepacia* K56-2 Δ*pglL.*	This study
B. cenocepacia K56-2 Δ*pglL*ΔmotB	Δ*pglL*ΔmotB derivative of K56-2 created from *B. cenocepacia* K56-2 Δ*pglL.*	This study

Supplementary Table 11: Plasmid list

Plasmid	Description	Source/ Reference
pRK2013	Helper plasmid, non-replicating in Burkholderia, ori_{colE1}, RK2 derivative, mob^+ tra^+, kanamycin resistant (Kan)	3
pGPI-SceI	ori_{R6K}, mob^+, Ω Tp^R, including an ISce-I restriction site. Trimethoprim resistant (Tmp^R)	4
pDAI-SceI-SacB	ori_{pBBR1}, P_{dhfr}, mob^+, expressing ISce-I and the negative selection marker SacB. Tetracycline resistant (Tet^R)	4,5
pSCrhaB2	ori_{pBBR1}, *rhaR, rhaS,* P_{rhaB}, mob^+. Trimethoprim resistant (Tmp^R)	6
pGPI-SceI-Δ*fliF*	pGPI-SceI containing fragments flanking *fliF* (BCAL0525) generated using Primers 0755 and 0756; Nsco_0753 and Nsco_0754. Trimethoprim resistant (Tmp^R)	This study
pGPI-SceI-BCAM0505	pGPI-SceI containing fragments flanking BCAM0505 generated using Primers 0759 and 0760; Nsco_0761 and Nsco_0762. Trimethoprim resistant (Tmp^R)	This study
pGPI-SceI-Δ*motB*	pGPI-SceI containing fragments flanking *motB* (BCAL0127) generated using Primers 0900 and 0901; Nsco_0902 and Nsco_0903. Trimethoprim resistant (Tmp^R)	This study
pSCrhaB2-*fliF*	pSCrhaB2 Rhamnose inducible plasmid containing C-terminal his_{10}-tagged *fliF* (BCAL0525) generated using primers Nsco_0331 and Nsco_0332. Trimethoprim resistant (Tmp^R)	This study
pSCrhaB2-$fliF_{S358A}$	pSCrhaB2 Rhamnose inducible plasmid containing C-terminal his_{10}-tagged *fliF* (BCAL0525) with an alanine substitution at position 358 generated using primers Nsco_0913 and Nsco_0914. Trimethoprim resistant (Tmp^R).	This study
pSCrhaB2-*motB*	pSCrhaB2 Rhamnose inducible plasmid containing C-terminal his_{10}-tagged *motB* (BCAL0127) using primers Nsco_0906 and Nsco_0907. Trimethoprim resistant (Tmp^R).	This study
pSCrhaB2-$motB_{S331A}$	pSCrhaB2 Rhamnose inducible plasmid containing C-terminal his_{10}-tagged *motB* (BCAL0127) with an alanine substitution at position 331 generated using primers Nsco_0915 and Nsco_0916. Trimethoprim resistant (Tmp^R).	This study

Supplementary Table 12: Primer list

Primer names	Description	Sequence
Nsco_0331	pScrhaB2 *fliF* (BCAL0525) forward	gaaattcagcaggatcacatatgGCATGGATTCGCAGGCCAACTCG
Nsco_0332	pScrhaB2 *fliF* (BCAL0525) reverse his-tag	catgcctgcaggtcgactctagatcaGTGGTGGTGGTGGTGGTGGTGGTGGTGGCGTTCATCGGACACCCAGTTC
Nsco_0753	Upstream_fwd – *B. cenocepacia* K56-2 for mutagenesis of *fliF* (BCAL0525)	gcatgcgatatcgagctctcccGCGCTTCTCCTTGTTATTGC
Nsco_0754	Upstream_rev - *B. cenocepacia* K56-2 for mutagenesis of *fliF* (BCAL0525)	GTTCGTAGCGGTTCTTCTCGTCAATCCATGCGTCGAGTTTCTC
Nsco_0755	Downstream_fwd - *B. cenocepacia* K56-2 for mutagenesis of *fliF* (BCAL0525)	GAGAAACTCGACGCATGGATTGACGAGAAGAACCGCTACGAAC
Nsco_0756	Downstream_rev - *B. cenocepacia* K56-2 for mutagenesis of BCAL0525	cggataacaatttgtggaattcccCAGGTTCTCGAACACGAACA
Nsco_0759	Upstream_fwd - *B. cenocepacia* K56-2 for mutagenesis of BCAM0505	gcatgcgatatcgagctctcccGATTCGACTTCGACCGTCAC
Nsco_0760	Upstream_rev - *B. cenocepacia* K56-2 for mutagenesis of BCAM0505	CTTCTTCACGGCGACCTTCTCATGTTCATTGATGTTCTCCTTCG
Nsco_0761	Downstream_fwd - *B. cenocepacia* K56-2 for mutagenesis of BCAM0505	CGAAGGAGAACATCAATGAACATGAGAAGGTCGCCGTGAAGAAG
Nsco_0762	Downstream_rev - *B. cenocepacia* K56-2 for mutagenesis of BCAM0505	cggataacaatttgtggaattcccTTGACTACGAAACGCTGCTG
Nsco_0809	BCAM0505 screening primer Fwd	GCTCGCCCATTCGGTCGCATC
Nsco_0810	BCAM0505 screening primer Rev	GGAAGTCGAGCGCGAAGGCTG
Nsco_0811	*fliF* (BCAL0525) screening primer Fwd	GCGCGCCCTGGTACAGCATC
Nsco_0812	*fliF* (BCAL0525) screening primer Rev	CGCTGCGACATGTTCGCGAGG
Nsco_0900	Upstream_Fwd – *B. cenocepacia* K56-2 for mutagenesis of *motB* (BCAL0127)	gcatgcgatatcgagctctcccGCTCTTCAAGGGCTCGAAAT
Nsco_0901	Upstream_Rev - *B. cenocepacia* K56-2 for mutagenesis of *motB* (BCAL0127)	TCAGCGCTTCTTCGGATTTATGAATCCTTGCTCTTGCTCATCG
Nsco_0902	Downstream_Fwd - *B. cenocepacia* K56-2 for mutagenesis of *motB* (BCAL0127)	CGATGAGCAAGAGCAAGGATTCATAAATCCGAAGAAGCGCTGA

Nsco_0903	Downstream_Rev - *B. cenocepacia* K56-2 for mutagenesis of *motB* (BCAL0127)	cggataacaatttgtggaattcccAACACGTCGACCATGTCCTT
Nsco_0904	*motB* (BCAL0127) screening primer Fwd	ACACTCGTGACGCTGTTGTC
Nsco_0905	*motB* (BCAL0127) screening primer Rev	CTTCAGCCGTTCGAGCTTC
Nsco_0906	pScrhaB2 *motB* (BCAL0127) Fwd	gaaattcagcaggatcacatatgAGCAAGAGCAAGGATCGCGCGATCG
Nsco_0907	pScrhaB2 *motB* (BCAL0127) his tag Rev	catgcctgcaggtcgactctagatcaGTGGTGGTGGTGGTGGTGGTGGTGGTGGGGTTTCGGCGCCACGGCGACGGC
Nsco_0913	pSCrhaB2 *fliF* (BCAL0525) his S358A Fwd	CGAACACGCCGCCGCAGCCGGCCGCCGCGCCGATCGTCGCCGGCAACGG
Nsco_0914	pSCrhaB2 *fliF* (BCAL0525) his S358A Rev	CCGTTGCCGGCGACGATCGGCGCGGCGGCCGGCTGCGGCGGCGTGTTCG
Nsco_0915	pSCrhaB2 *motB* (BCAL0127) his S331A Fwd	GGTGCGTCCGGTCGTGGCGGCCGCCGTCGCCGTGGCGCCGAAACC
Nsco_0916	pSCrhaB2 *motB* (BCAL0127) his S331A Rev	GGTTTCGGCGCCACGGCGACGGCGGCCGCCACGACCGGACGCACC

Supplementary Table 13: Proteomic Dataset

Pride accession number (Review login details)	MS instrument	Number of Biological groups, replicates and total datafiles	Description of dataset
PXD043184<br>Username:<br>reviewer_pxd043184@ebi.ac.uk<br>Password:<br>8EGoWAob	Orbitrap Fusion Eclipse	3 biological group, 4 replicates total of 12 datafiles	DDA experiment confirming generation of two independent Δ*BCAM0505* strains compared to WT K56-2 at stationary phase within LB.
PXD043186<br>Username:<br>reviewer_pxd043186@ebi.ac.uk<br>Password:<br>mPr2COOG	Orbitrap Q-Exactive plus	3 biological group, 4 replicates total of 12 datafiles	DDA experiment confirming generation of two independent Δ *fliF* (BCAL0525) strains compared to WT K56-2 at stationary phase within LB.
PXD043187<br>Username:<br>reviewer_pxd043187@ebi.ac.uk<br>Password: yyFKZi4V	Orbitrap Fusion Lumos	3 biological group, 4 replicates total of 12 datafiles	DDA experiment confirming generation of two independent *ΔmotB* (BCAL0127) strains compared to WT K56-2 at stationary phase within LB.
PXD043188<br>Username:<br>reviewer_pxd043188@ebi.ac.uk<br>Password: vxk0CARM	Orbitrap Fusion Lumos	1 biological group, 3 growth media 4 replicates total of 12 datafiles	FAIMS analysis of the *B. cenocepacia* K56-2 glycoproteome at mid-log phase across LB, TSB and ASM media.
PXD043193<br>Username:<br>reviewer_pxd043193@ebi.ac.uk<br>Password: lHgqdtlM	Orbitrap Fusion Lumos	1 biological group, 3 growth media 4 replicates total of 12 datafiles	FAIMS analysis of the *B. cenocepacia* K56-2 glycoproteome at stationary phase across LB, TSB and ASM media.
PXD043194<br>Username:<br>reviewer_pxd043194@ebi.ac.uk<br>Password:<br>8dv03UQC	Orbitrap Fusion Lumos	1 biological group, 3 growth media 4 replicates*2, total of 24 datafiles	ZIC-HILIC enrichment of *B. cenocepacia* K56-2 glycopeptides from across LB, TSB and ASM grown cells.

PXD043197<br>Username:<br>reviewer_pxd043197@ebi.ac.uk<br>Password:<br>UrI1g3p5	Orbitrap Fusion Eclipse	5 biological group, 4 replicates total of 20 datafiles	DIA experiment to assess proteomic impacts of glycoprotein mutants (Δ*fliF,* Δ*motB,* ΔBCAM0505) compared to WT K56-2 and Δ*pglL* at stationary phase within LB.
PXD043221<br>Username:<br>reviewer_pxd043221@ebi.ac.uk<br>Password: J80YUL3o	Orbitrap Fusion Eclipse	1 biological group, 3 growth media 4 replicates at two growth phases (log & stationary), total of 24 datafiles	DIA experiment to assess alterations within glycoprotein levels in WT K56-2 across the proteome at log and stationary phase across LB, TSB and ASM media.
PXD043227<br>Username:<br>reviewer_pxd043227@ebi.ac.uk<br>Password: TLwfYac8	Orbitrap Fusion Eclipse	3 biological group, 3 growth media 4 replicates, total of 36 datafiles	DIA experiment to assess alterations within glycoproteins levels across the proteome at stationary phase in LB, TSB and ASM media within WT, Δ*pglL* and Δ*pglL*Δ*amrAB::native-pglL-*his_{10}.
PXD043280<br>Username:<br>reviewer_pxd043280@ebi.ac.uk<br>Password: 6irwg83X	Orbitrap Fusion Eclipse	6 biological group, 2 growth media (LB -/+ 0.1% Rhamnose) 4 replicates, total of 48 datafiles	DIA experiment to assess the impact of MotB complementation within WT and Δ*motB* strains at stationary phase within LB.
PXD045246<br>Username:<br>reviewer_pxd045246@ebi.ac.uk<br>Password: netlyV4R	Orbitrap Fusion Eclipse	4 biological group, 2 growth media (LB -/+ 0.1% Rhamnose) 4 replicates, total of 32 datafiles	DIA experiment to assess the impact of FliF complementation within Δ*fliF* strains containing pSCrhaB2 vectors compared to WT containing pSCrhaB2 empty vector at stationary phase within LB.

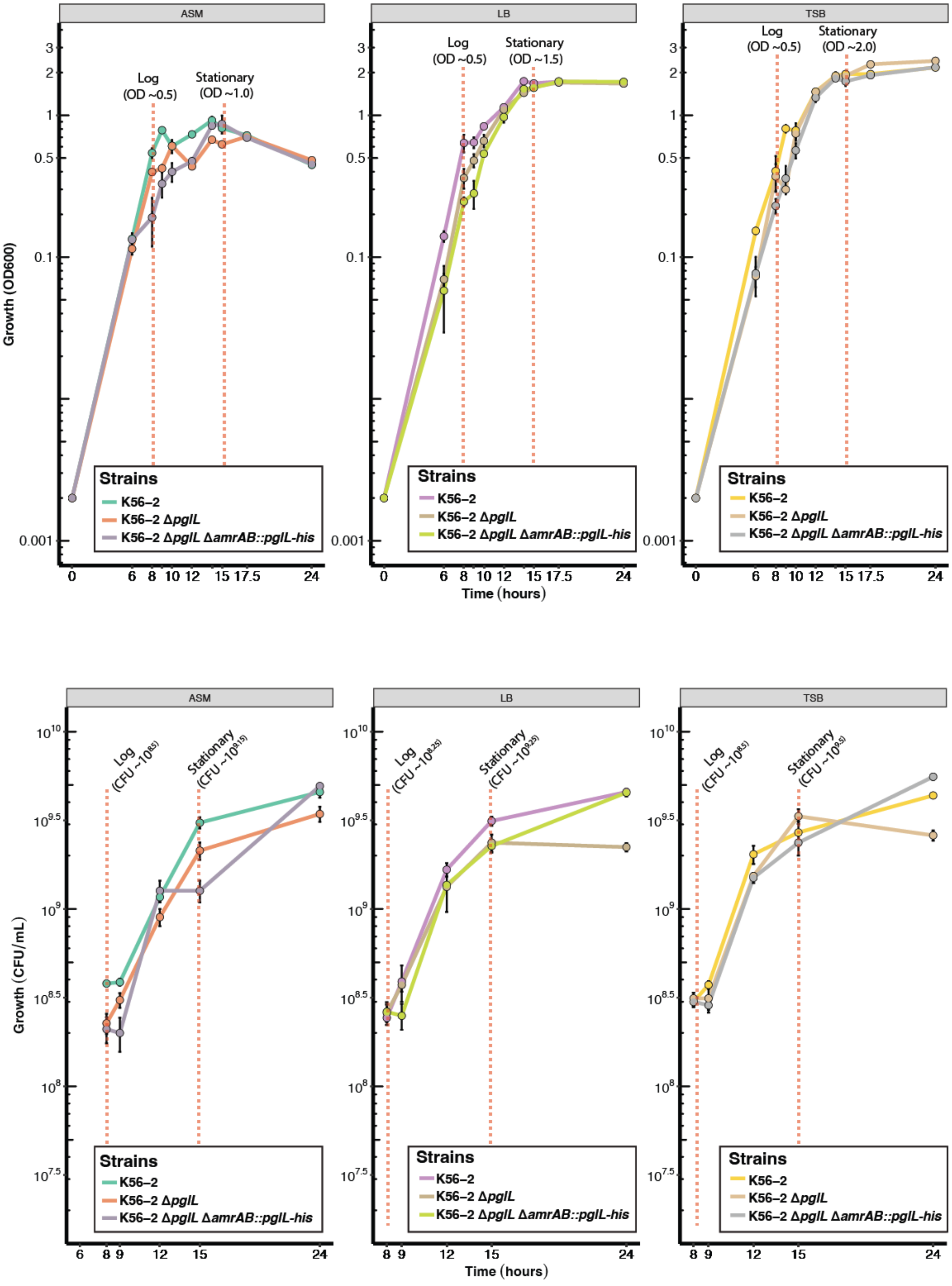

Supplementary Figure 1. Growth curves and CFU per mL counts of strains across the different media used within this study. Dotted lines denote growth collection points.

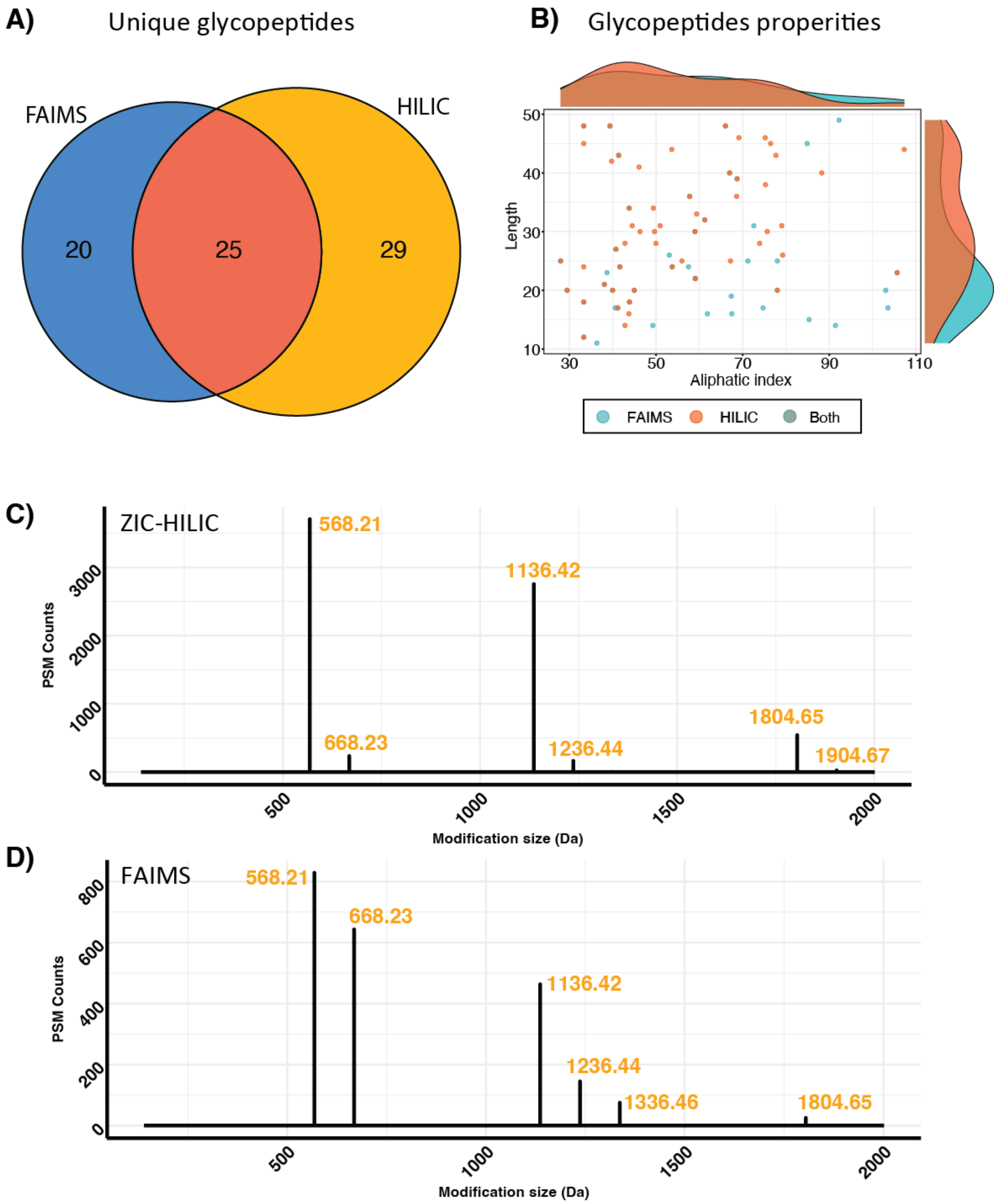

Supplementary Figure 2. Comparison of ZIC-HILIC and FAIMS glycopeptide enrichment approaches. A) Venn diagram of the unique glycopeptides identified across ZIC-HILIC and FAIMS enrichments used in this study reveals both enrichment approaches access unique glycopeptides**. B)** Comparison of glycopeptide size and aliphatic properties reveals differences in the physiochemical properties of glycopeptides enriched using ZIC-HILIC and FAIMS as previously reported. **C)** Distribution of glycans observed on glycopeptides reveals similar glycoforms distributions between glycopeptide enrichment approaches yet a greater number of glyco-PSMs are identified using ZIC-HILIC.

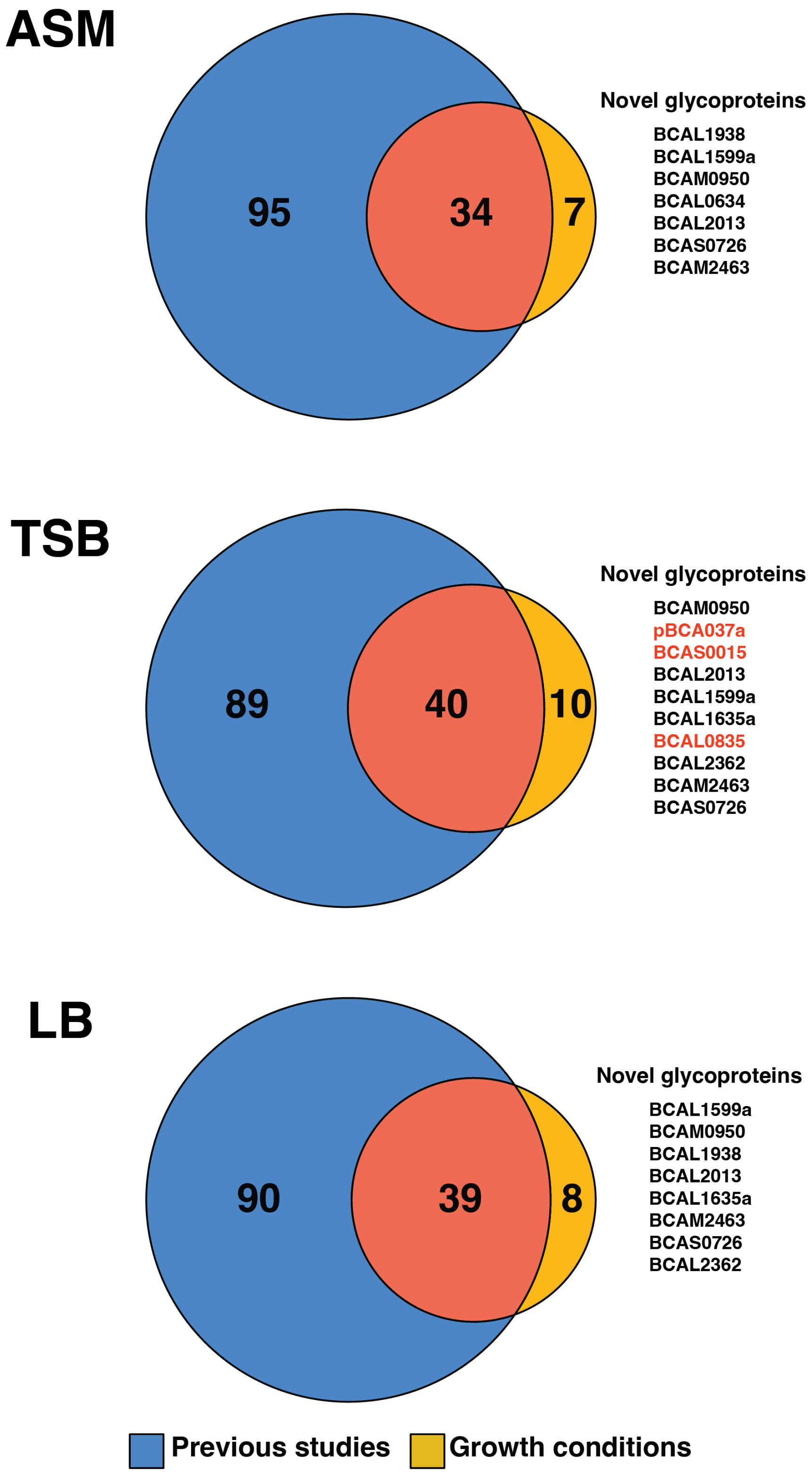

Supplementary Figure 3. Unique glycoproteins identified within this study across different growth media. Comparisons of glycoproteins identified across each media with previous studies (129 known glycoproteins) reveal multiple novel glycoproteins are observed within different growth conditions. Glycoproteins unique to a single growth media are denoted in red.

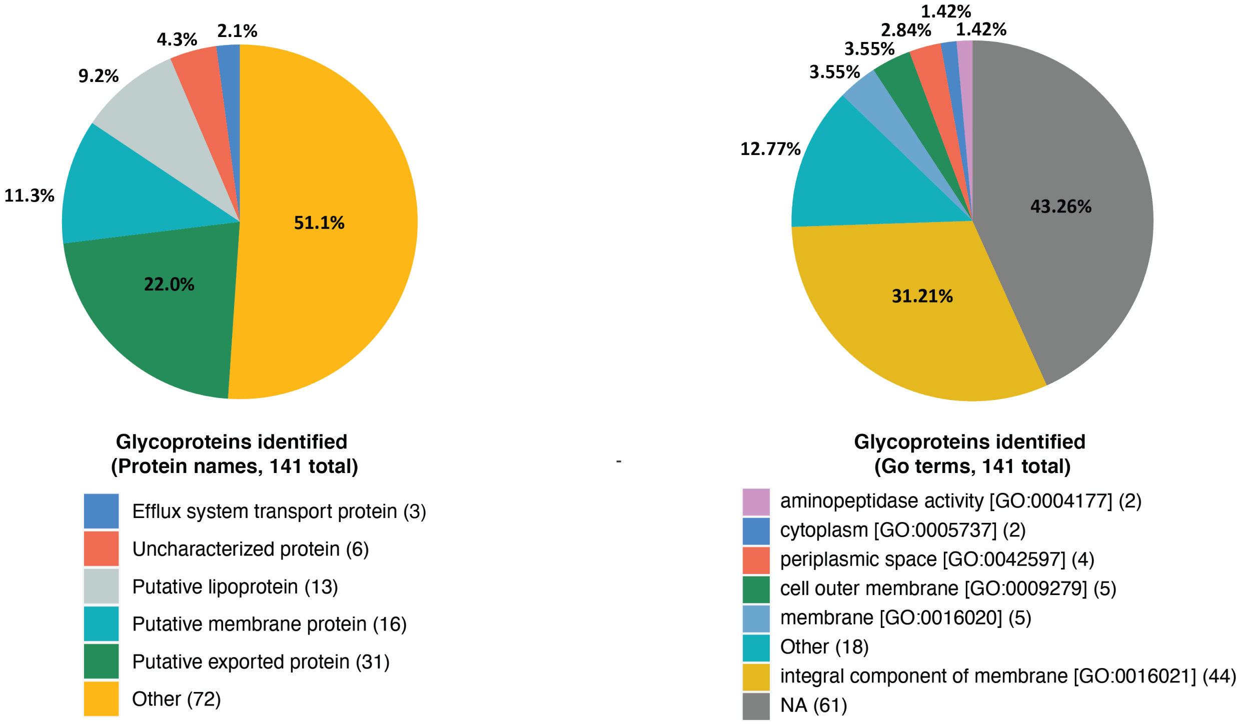

Supplementary Figure 4. *B. cenocepacia* glycoproteome GO term and functional assignments. >40% of glycoproteins are defined as putative or uncharacterised proteins and >40% lack any assigned GO terms within Uniprot.

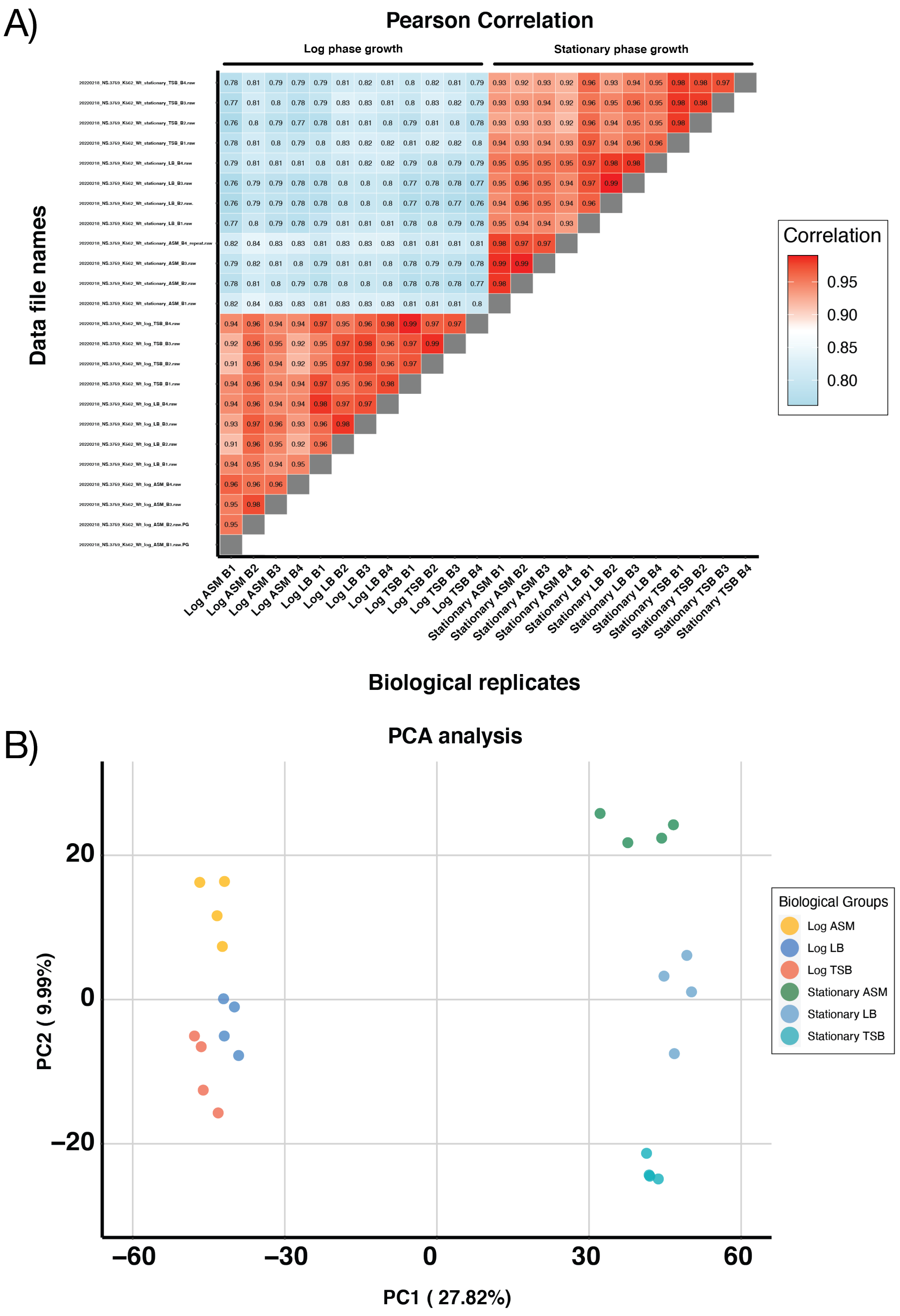

Supplementary Figure 5. Pearson correlation and PCA analysis of *B. cenocepacia* proteomes across growth phases within different media. A) Pearson correlation analysis reveals samples have high correlation (>0.90) within individual growth conditions. **B)** PCA analysis reveals each biological condition clusters with its corresponding group based on growth media and growth phase with growth phase driving separation along the PC1 and media type driving separation along the PC2.

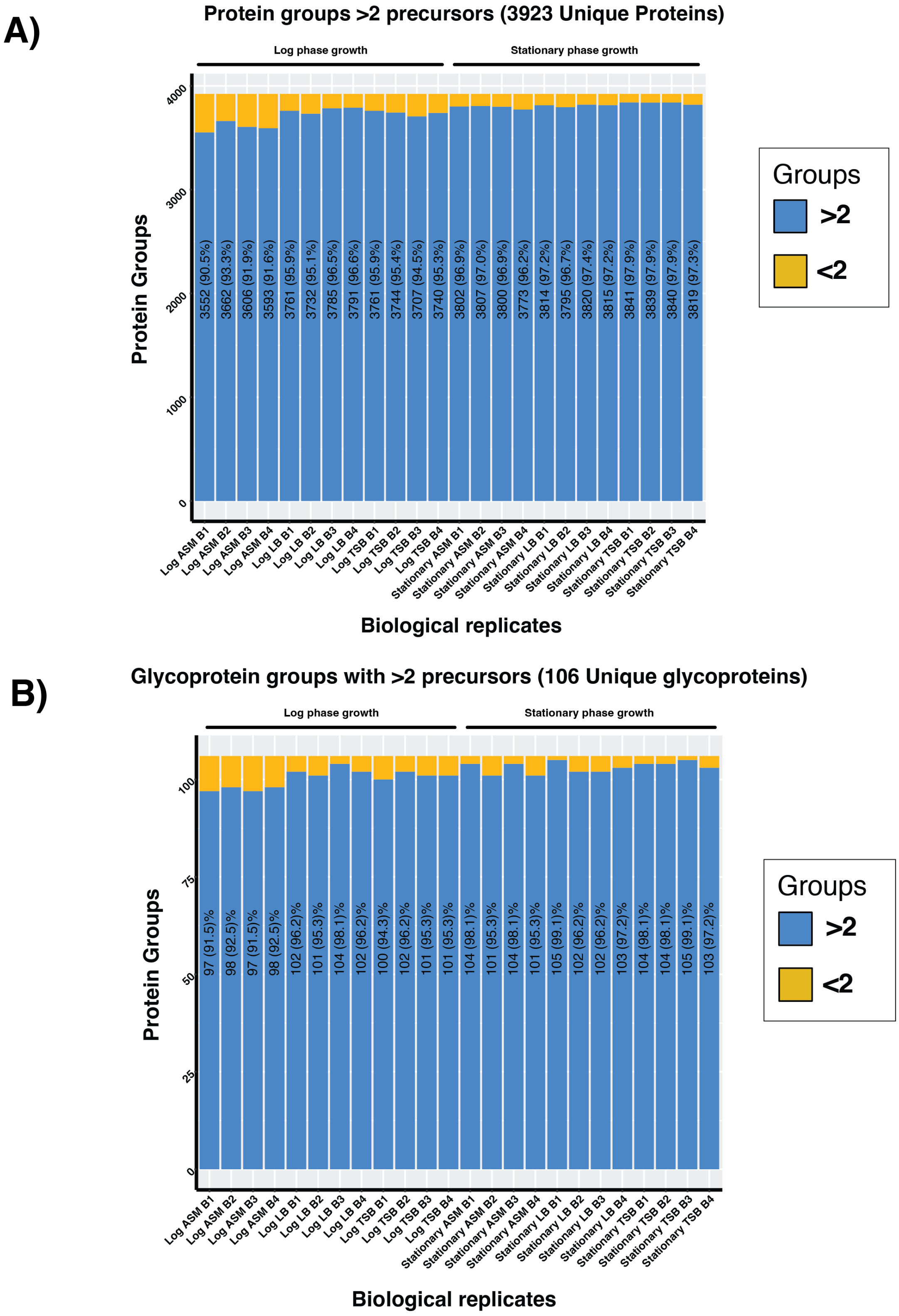

Supplementary Figure 6. Precursor coverage within *B. cenocepacia* proteomes across growth phases and different media. >90% of proteins (A) and glycoproteins (B) are identified with greater than two precursors.

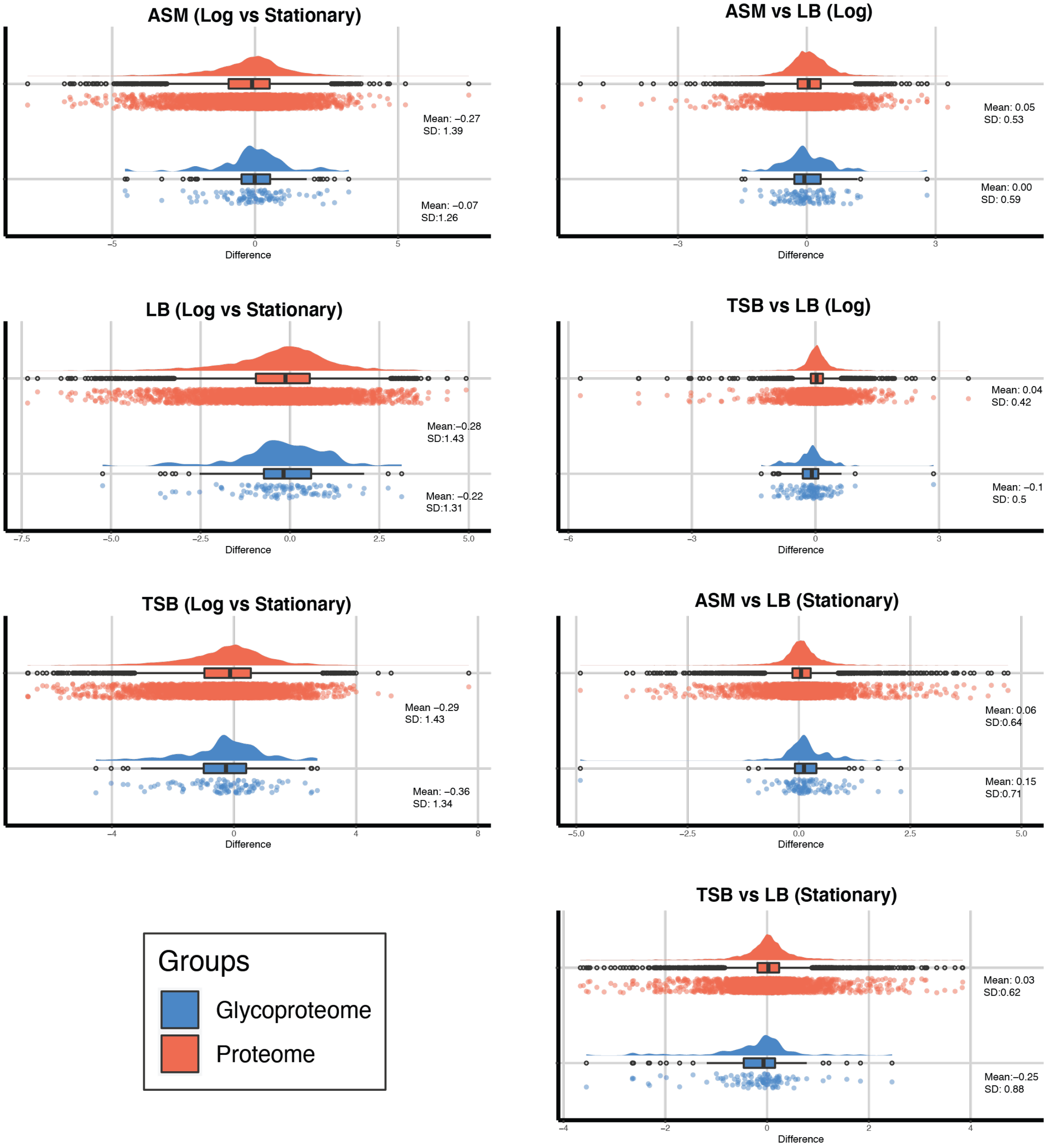

Supplementary Figure 7. Observed fold changes within the *B. cenocepacia* Proteome and glycoproteome across growth phases and different media. The glycoproteome and proteome undergo similar alterations across growth phases and different media conditions.

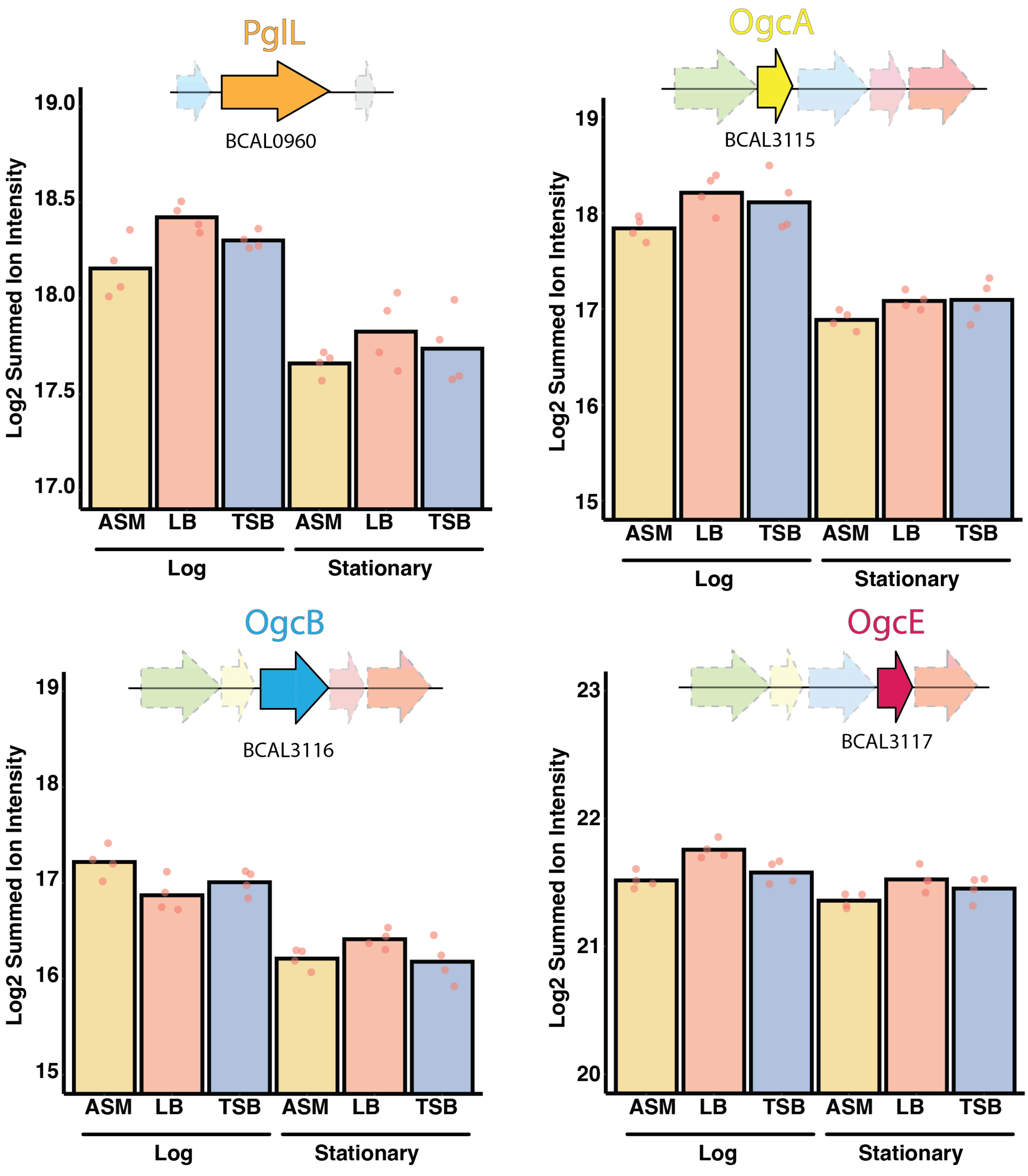

Supplementary Figure 8. Protein levels of observed glycosylation machinery across growth phases and different media. Across the proteins PglL (BCAL0960), OgcA (BCAL3115), OgcB (BCAL3116) and OgcE (BCAL3117) minimal abundance changes are observed (≤1 log2) between conditions.

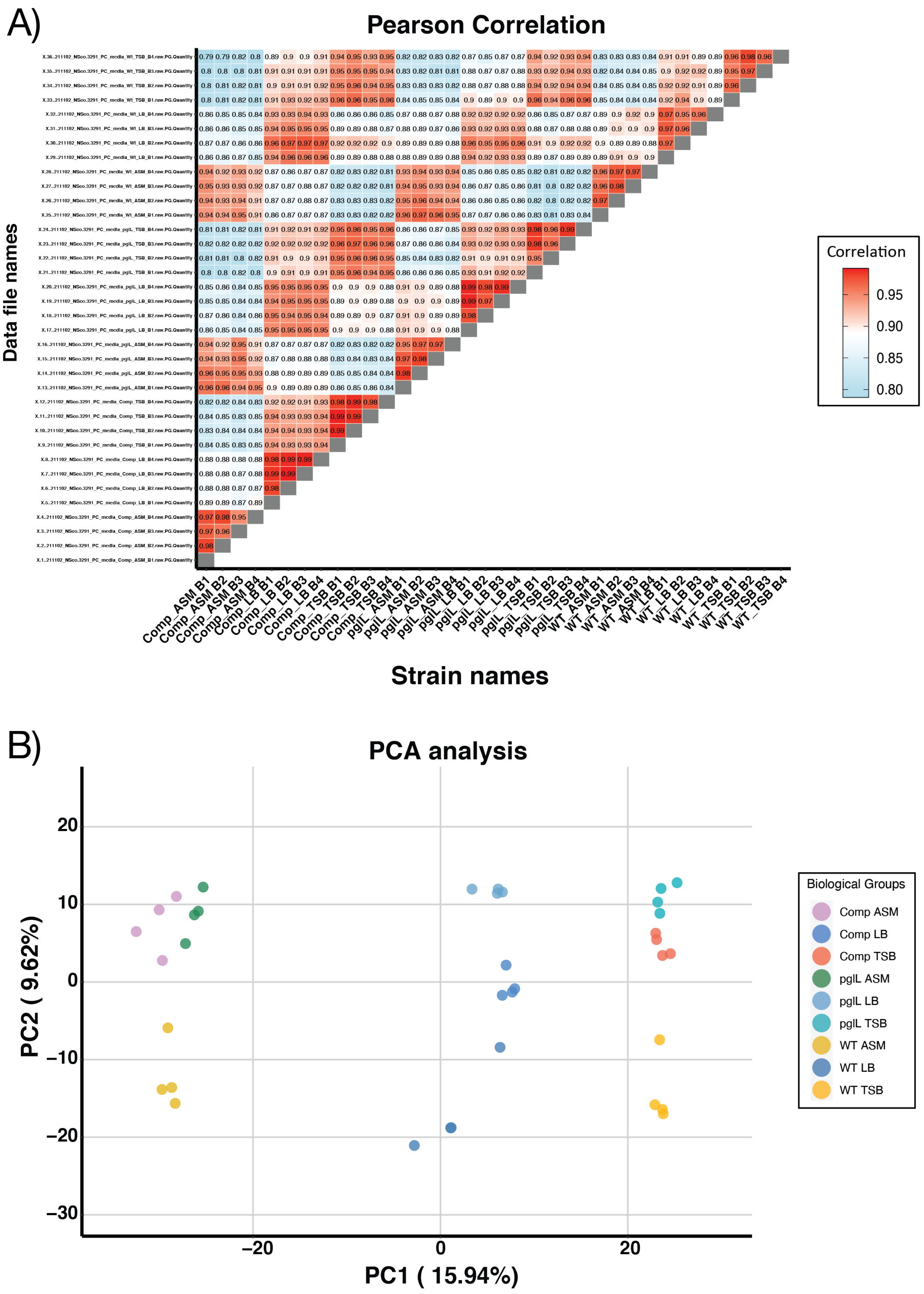

Supplementary Figure 9. Pearson correlation and PCA analysis of *B. cenocepacia* proteomes differing in the presence of glycosylation across growth phases. A) Pearson correlation analysis reveals samples have high correlation (>0.90) within individual growth conditions. **B)** PCA analysis reveals each biological condition clusters with its corresponding group based on growth media and growth phase with growth phase driving separation along the PC1 and media type driving separation along the PC2.

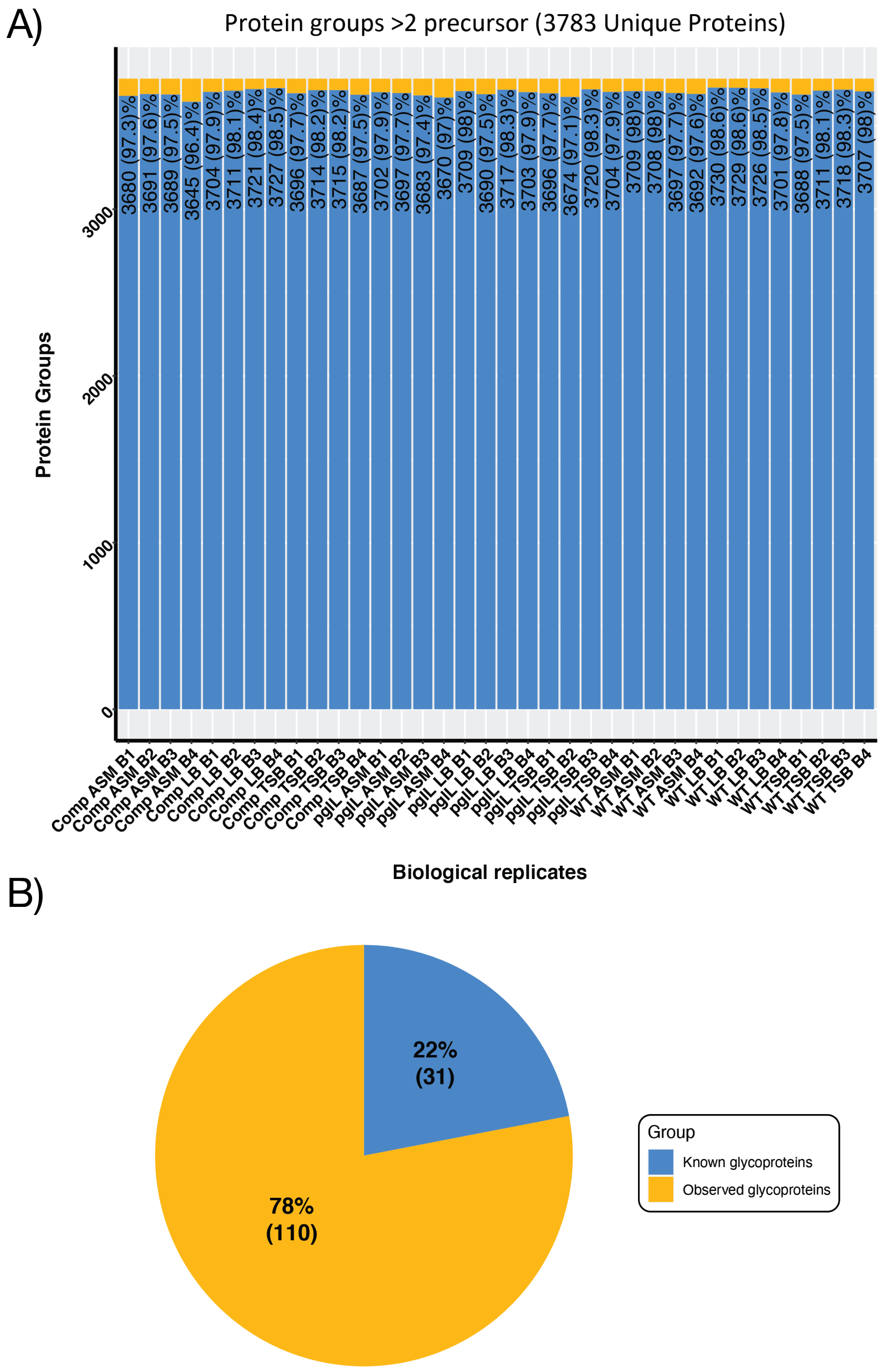

Supplementary Figure 10. Coverage of the *B. cenocepacia* proteome observed across different media and coverage of the known glycoproteome. A) A total of 3783 proteins are observed with >97% identified with multiple precursors in each replicate. **B)** 110 glycoproteins out of 141 were identified across replicates.

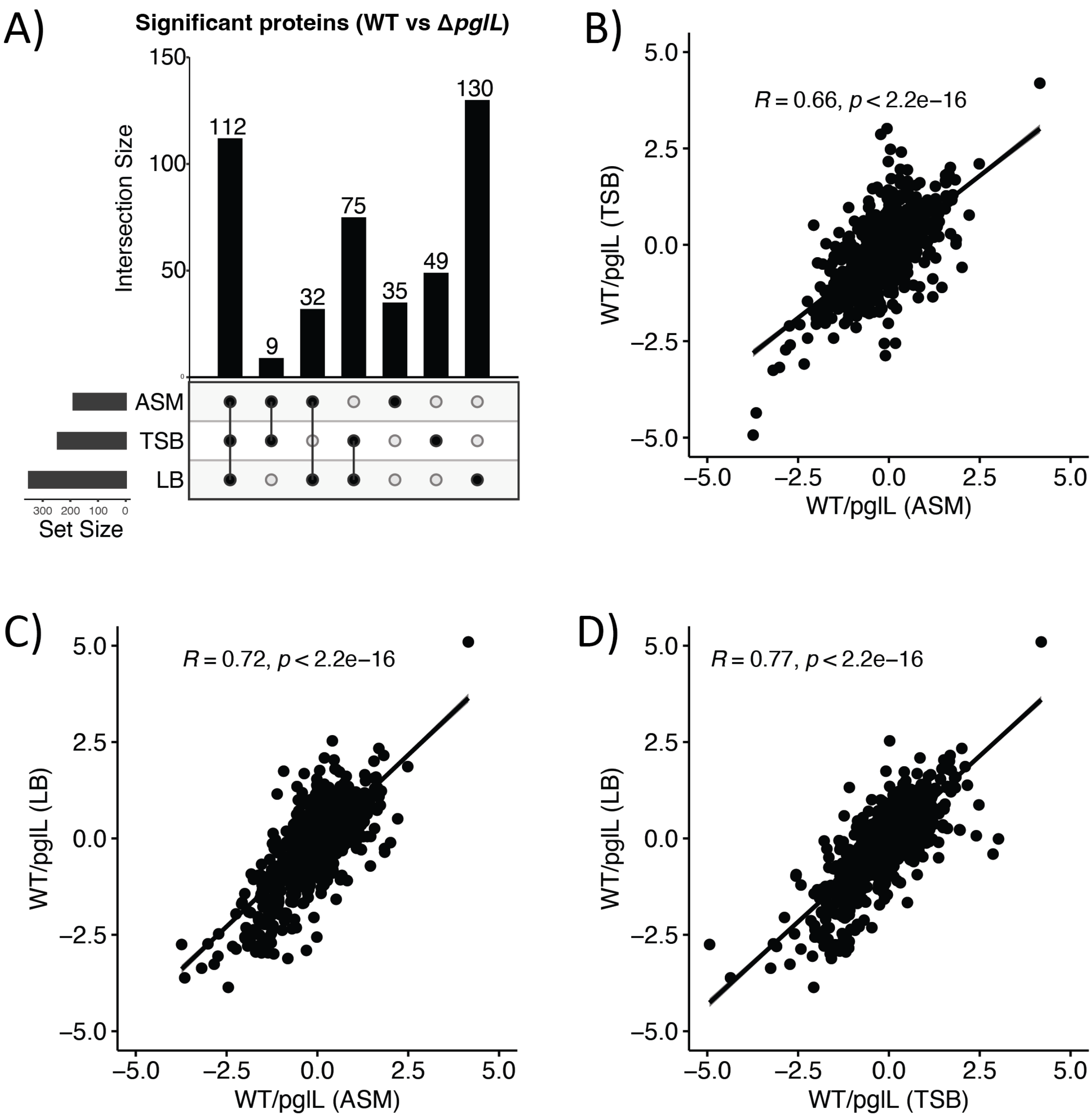

Supplementary Figure 11. Proteome changes observed within Δ*pglL* compared to WT under different growth phases. A) Upset plots of 442 proteins determined to be significantly altered within Δ*pglL* strains compared to WT across the growth conditions reveal >50% of all changes are observed across at least two growth media. **B to D)** Correlation (Pearson Correlation) analysis of the ratio of WT to Δ*pglL* across different growth media reveals highly similar changes regardless of growth media.

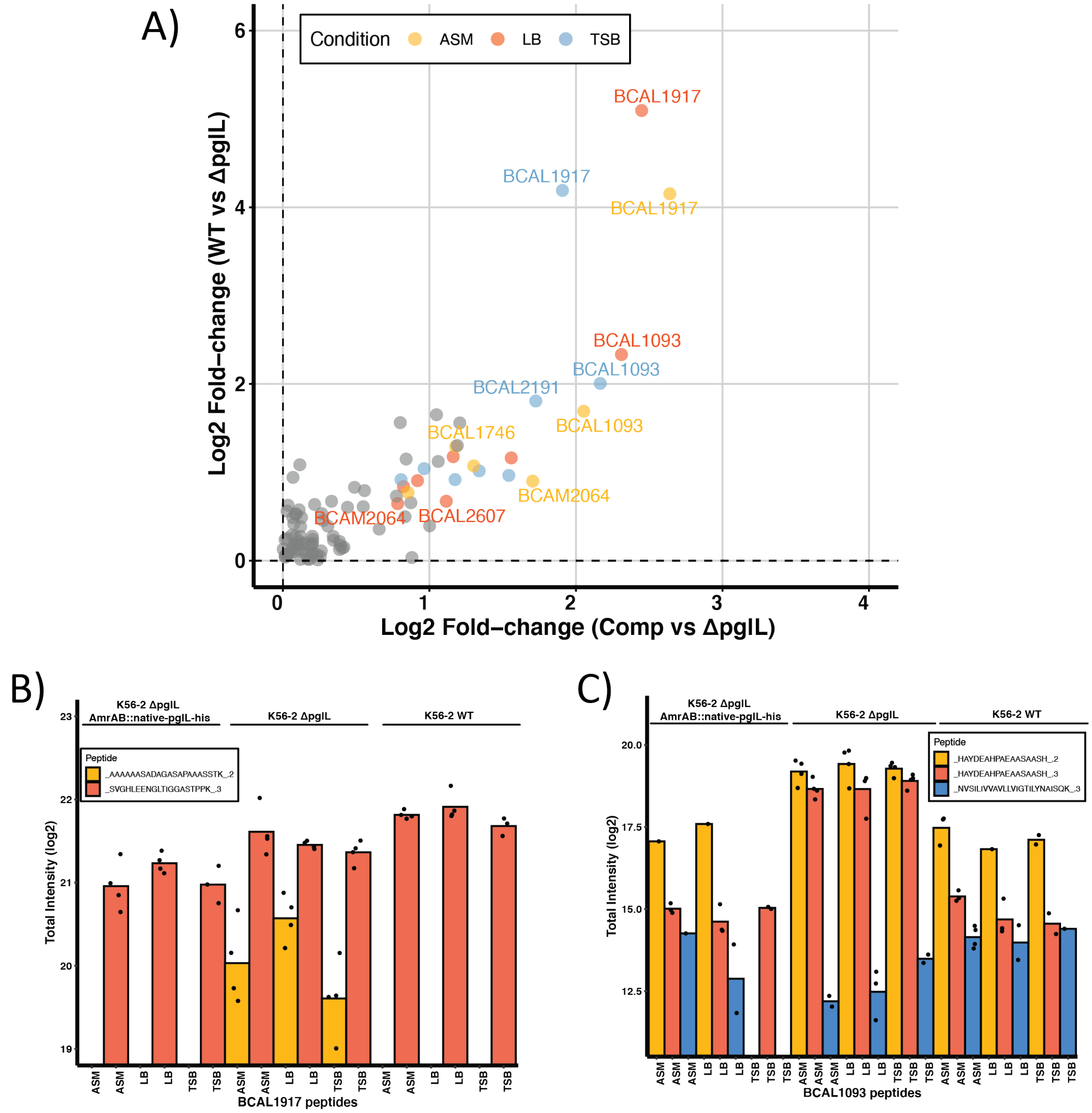

Known glycopeptide:

AAAAAASADAGASAPAAASSTK

Known glycopeptide:

HAYDEAHPAEAASAASH

Supplementary Figure 12. Analysis of *B. cenocepacia* glycoproteins observed to increase in abundance using DIA analysis. A) Scatter plot of observed glycoproteins abundances comparing the ratio of glycoprotein observed within the Complement vs Δ*pglL* (x-axis) and WT vs Δ*pglL* (y-axis). Coloured proteins correspond to proteins with a *p*-value of -log10(p-value) >2 in either comparison. Two proteins are observed to increase in abundance all growth media, BCAL1917 and BCAL1093. **B and C)** Analysis of the peptides associated with altered glycoproteins reveals the loss of glycosylation results in the appearance, in the case of BCAL1917, and increase in abundance, in the case of BCAL1093, of unmodified forms of peptides observed to be glycosylated (Supplementary Table 1) supporting the observed increase is an artefact resulting from the summation of peptide quantitation intensities into protein quantitation intensities.

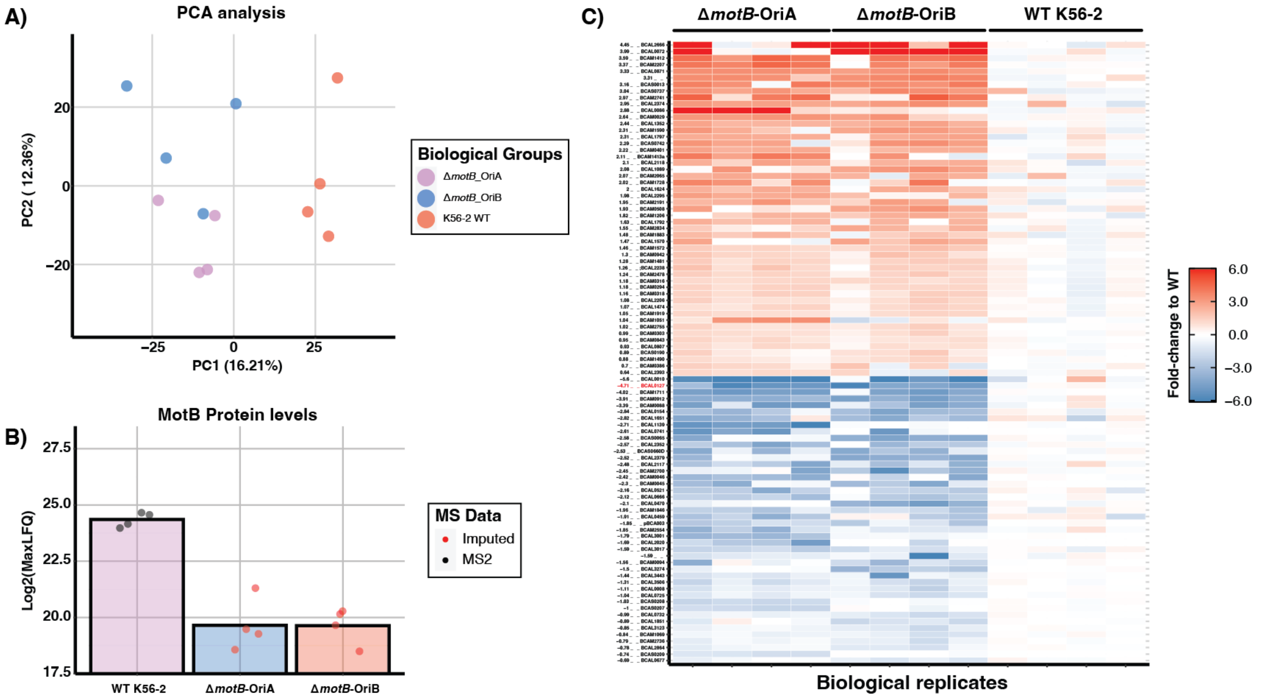

Supplementary Figure 13. Confirmation of *B. cenocepacia* Δ*motB* (BCAL0127) strains using DDA analysis. A) PCA analysis reveals each biological condition clusters with its corresponding group with strains being separated along the PC1. **B)** LFQ analysis of MotB reveals the loss of *motB* within mutant strains with MS2 values derived from imputed values. **C)** Heatmap of proteome changes observed within ΔBCAL0127 strains supports independent strains lead to similar proteomic alterations compared to WT. Proteomic results associated with analysis provided in Supplementary Table 5.

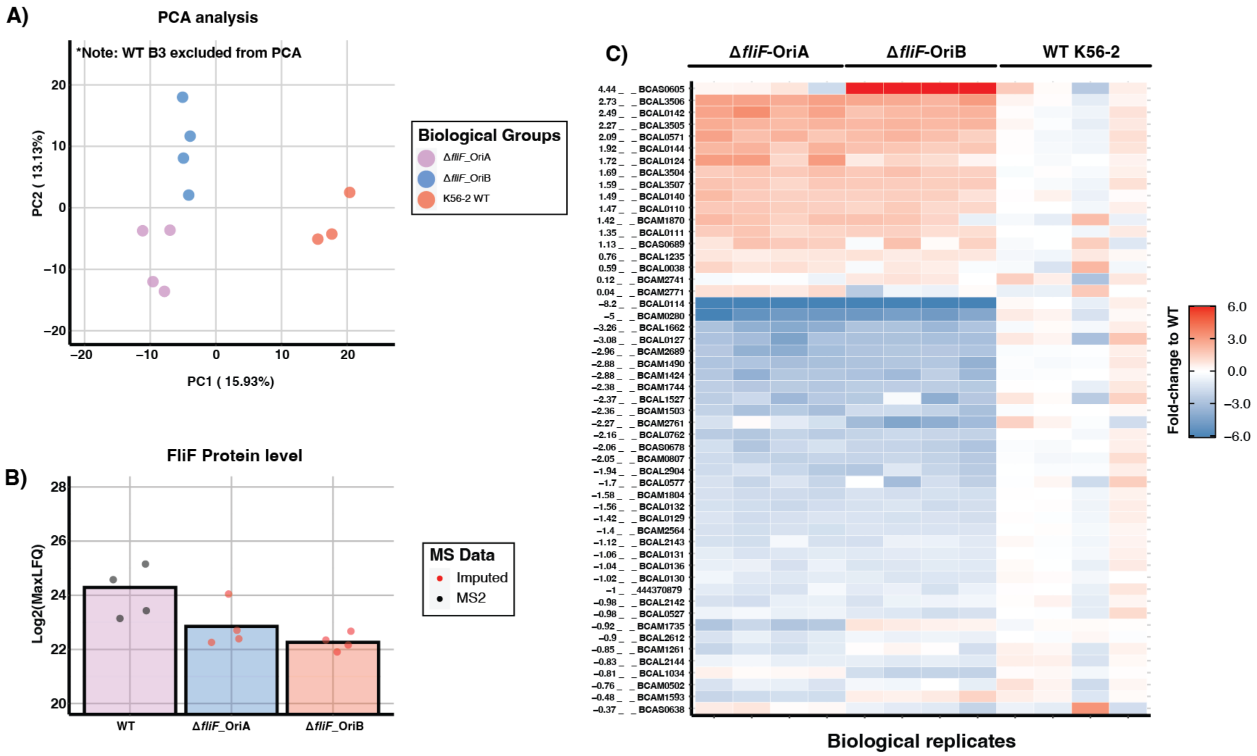

Supplementary Figure 14. Confirmation of *B. cenocepacia* Δ*fliF (*BCAL0525) strains using DDA analysis. A) PCA analysis reveals each biological condition clusters with its corresponding group with strains being separated along the PC1. **B)** LFQ analysis of FliF reveals the loss of *fliF* within mutant strains with MS2 values derived from imputed values. **C)** Heatmap of proteome changes observed within Δ*fliF* strains supports independent strains lead to similar proteomic alterations compared to WT. Proteomic results associated with analysis provided in Supplementary Table 5.

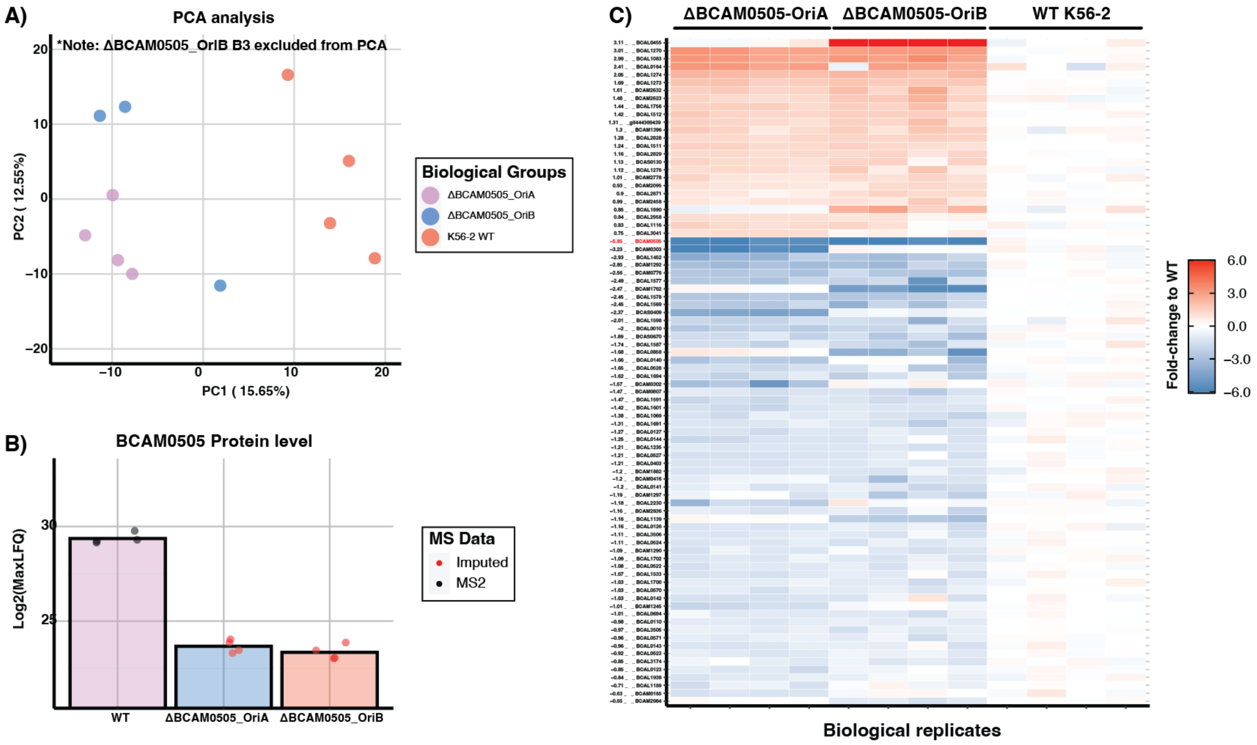

Supplementary Figure 15. Confirmation of *B. cenocepacia* Δ*BCAM0505* strains using DDA analysis. A) PCA analysis reveals each biological condition clusters with its corresponding group with strains being separated along the PC1. **B)** LFQ analysis of BCAM0505 reveals the loss of BCAM0505 within mutant strains with MS2 values derived from imputed values. **C)** Heatmap of proteome changes observed within Δ*BCAM0505* strains supports independent strains lead to similar proteomic alterations compared to WT. Proteomic results associated with analysis provided in Supplementary Table 5.

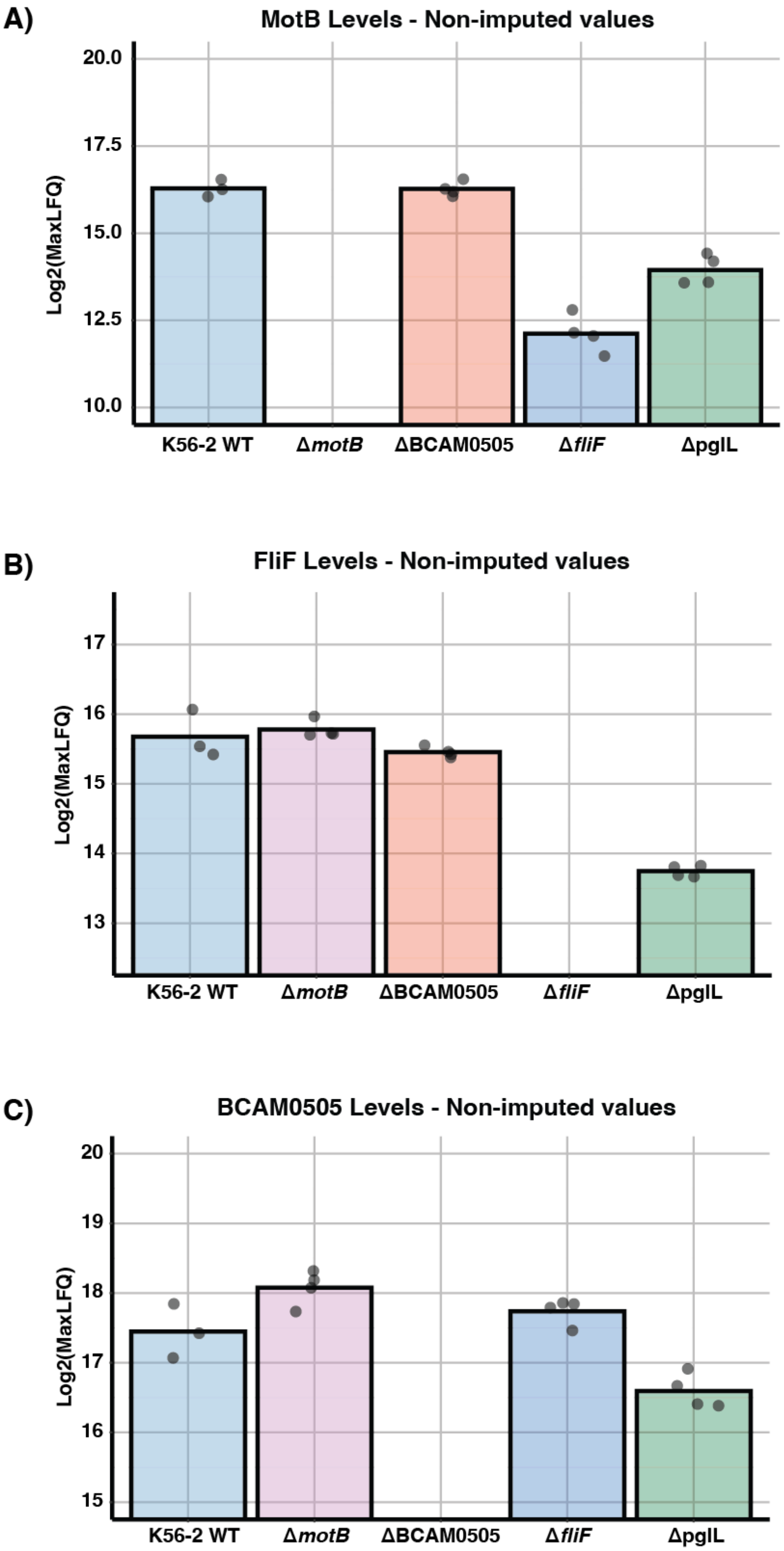

Supplementary Figure 16. Protein levels of MotB, FliF, and BCAM0505 across strains. DIA analysis confirms the absence of BCAL0127, BCAL0525, and BCAM0505 within strains.

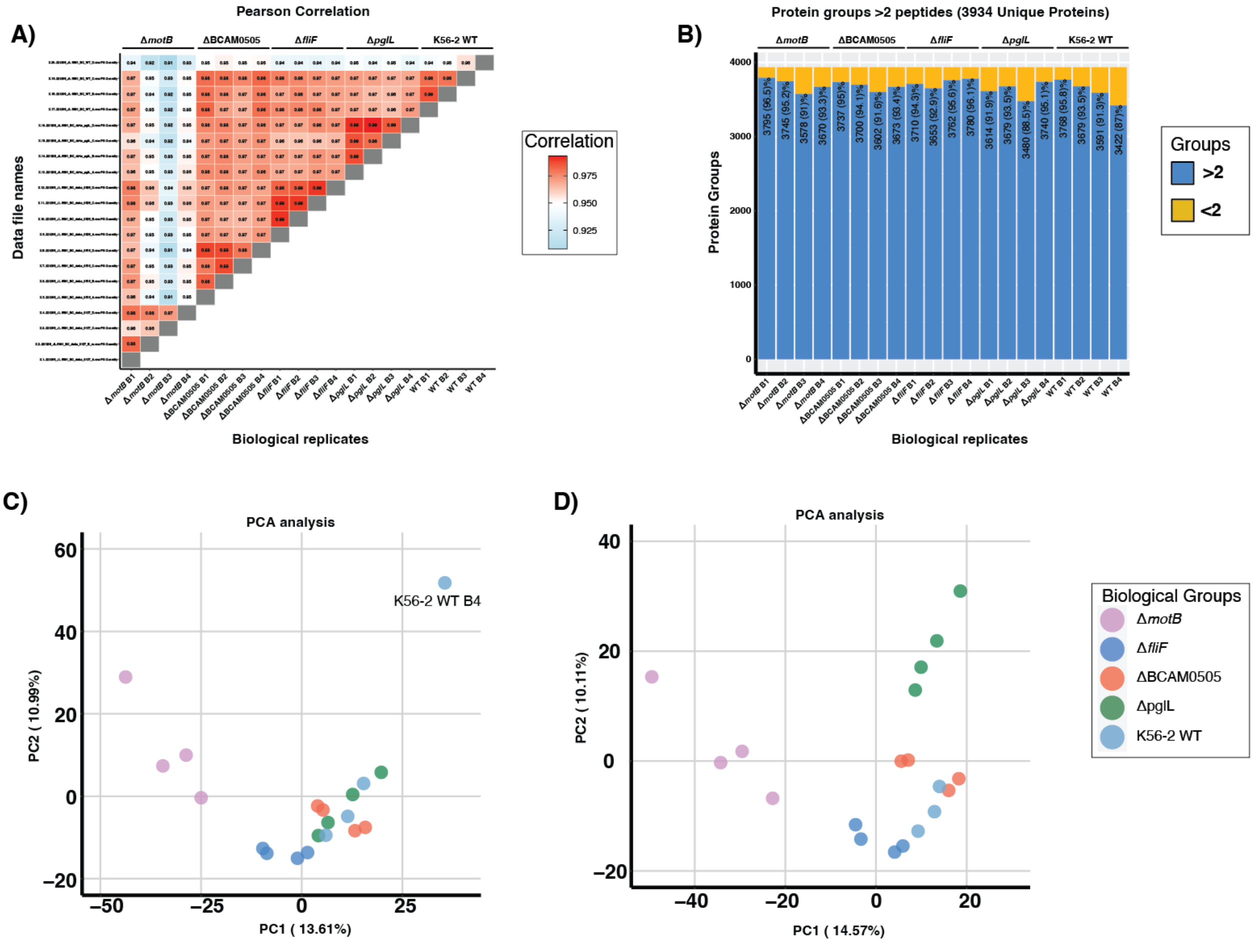

Supplementary Figure 17. QC plots of Δ*motB*, Δ*fliF*, Δ*BCAM0505,* WT and Δ*pglL* proteomes assessed using DIA analysis. A) Pearson correlation analysis reveals samples have high correlation (>0.90) within individual growth conditions. **B)** A total of 3934 proteins are observed with >90% of these proteins identified with multiple precursors within each replicate. **C)** PCA analysis reveals each biological condition clusters with its corresponding group except for a single replicate (WT B4). **D)** PCA analysis without WT B4 improves clustering of biological groups.

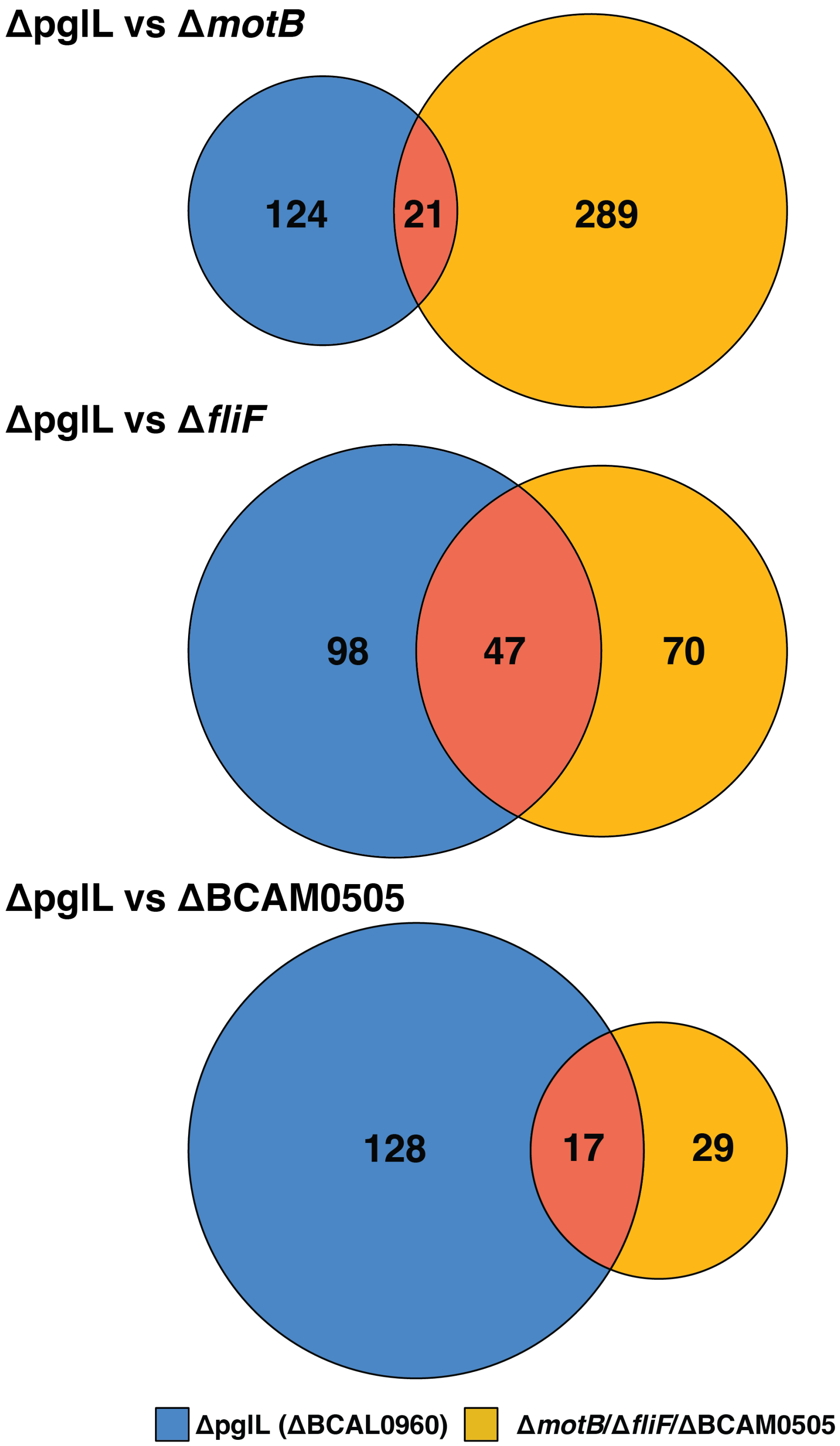

Supplementary Figure 18. Overlap in proteins altered observed in Δ*motB*, Δ*fliF*, ΔBCAM0505 versus WT compared to Δ*pglL* versus WT. Comparisons of proteins observed altered within Δ*pglL,* Δ*motB*, Δ*fliF*, and ΔBCAM0505 compared to WT.

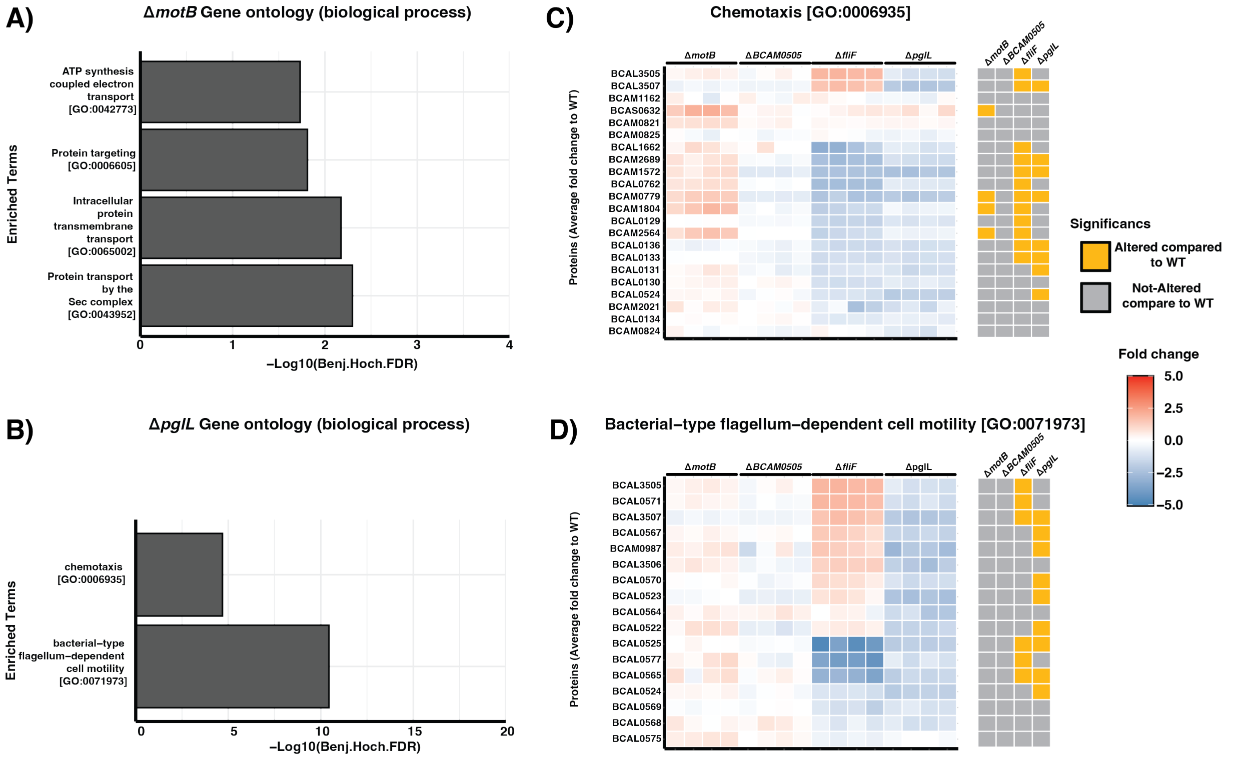

Supplementary Figure 19. Enrichment analysis of Gene Ontology (GO) terms associated with altered proteins in Δ*motB*, Δ*fliF*, ΔBCAM0505, Δ*pglL* versus WT. Enrichment analysis of Gene ontology (biological process) terms reveals the enrichment of functional terms within proteins observed altered within **A**) Δ*motB* compared to WT and **B**) Δ*pglL* compared to WT yet these are unique from each other. Heatmap of fold changes in proteins associated with the GO terms. **C)** Chemotaxis [GO:0006935] and **D)** Bacterial−type flagellum−dependent cell motility [GO:0071973]. Protein changes considered significant and used for enrichment analysis are denoted in Orange.

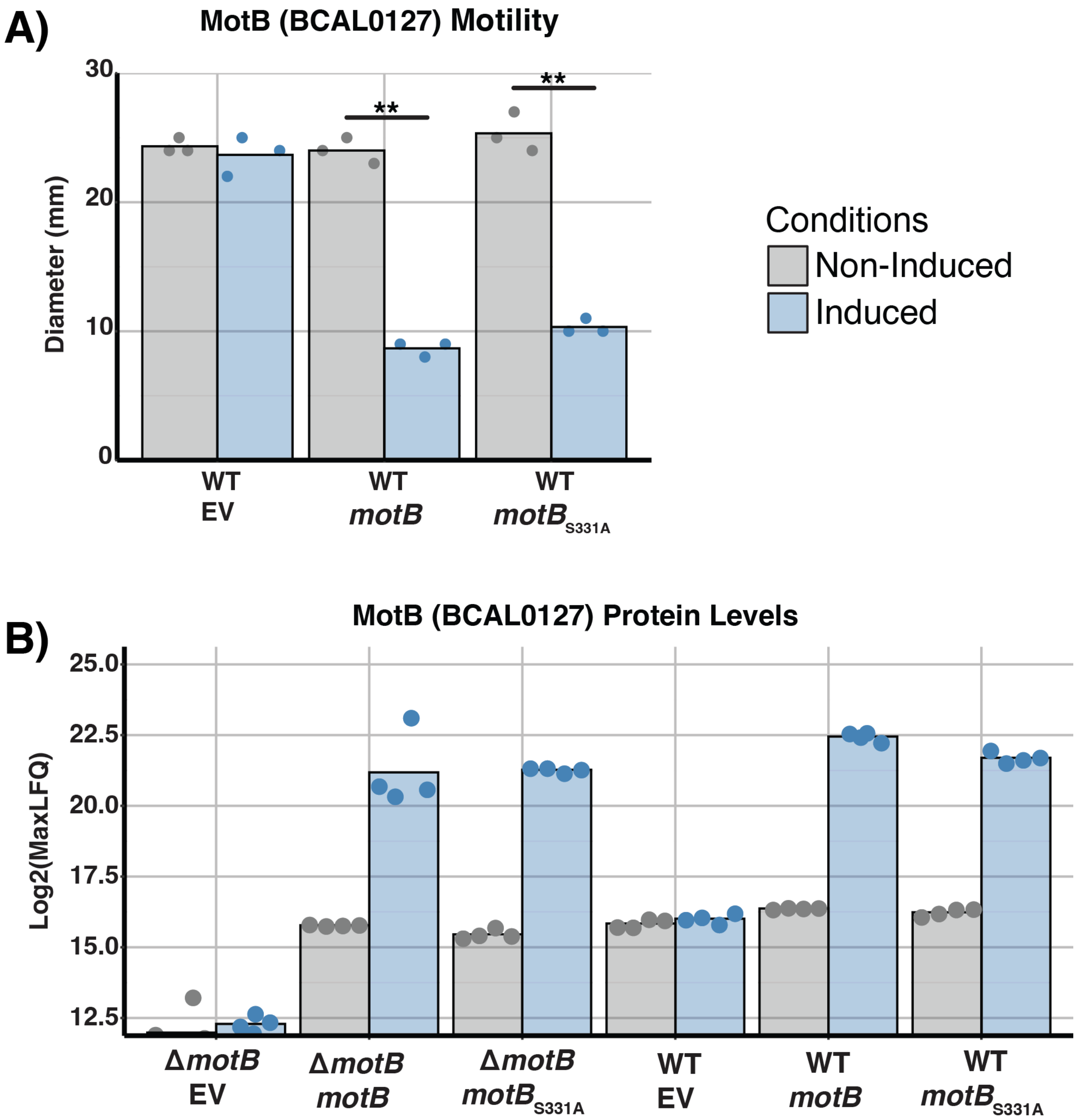

Supplementary Figure 20. Overexpression of *motB* from pSCrhaB2 leads to motility defects in *B. cenocepacia* K56-2. A) Motility assays demonstrate the induction of *motB* expression with rhamnose leads to reductions in motility within *B. cenocepacia k56-2* WT. **B)** MotB protein levels determined by DIA proteomic analysis revealed the induction of *motB* with 0.1% rhamnose results to a 5 log2(fold) or ~x30 increase in MotB levels with the lower boundaries of the MaxLFQ shown defined by the imputed values observed within Δ*motB* EV respectively.

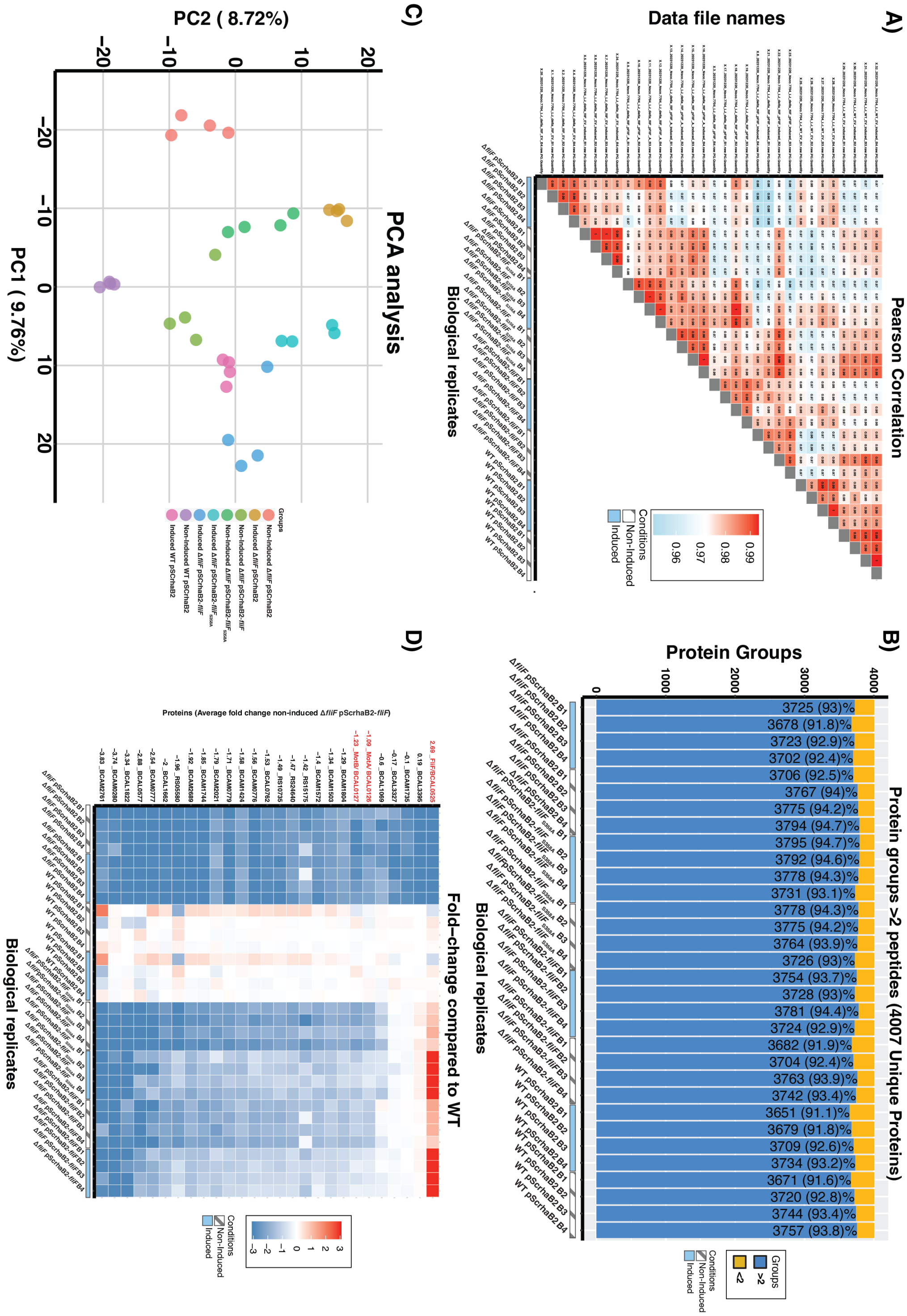

Supplementary Figure 21. QC plots of DIA analysis of *B. cenocepacia* strains containing *fliF* complementation vectors and controls. A) Pearson correlation analysis reveals samples have high correlation (>0.90) within individual growth conditions. **B)** A total of 4285 proteins are observed with >90% of these proteins identified with multiple precursors within each replicate. **C)** PCA analysis reveals each biological condition clusters with its corresponding group. **D)** MaxLFQ values for FliF across samples confirm the restoration of FliF in an inducible manner.

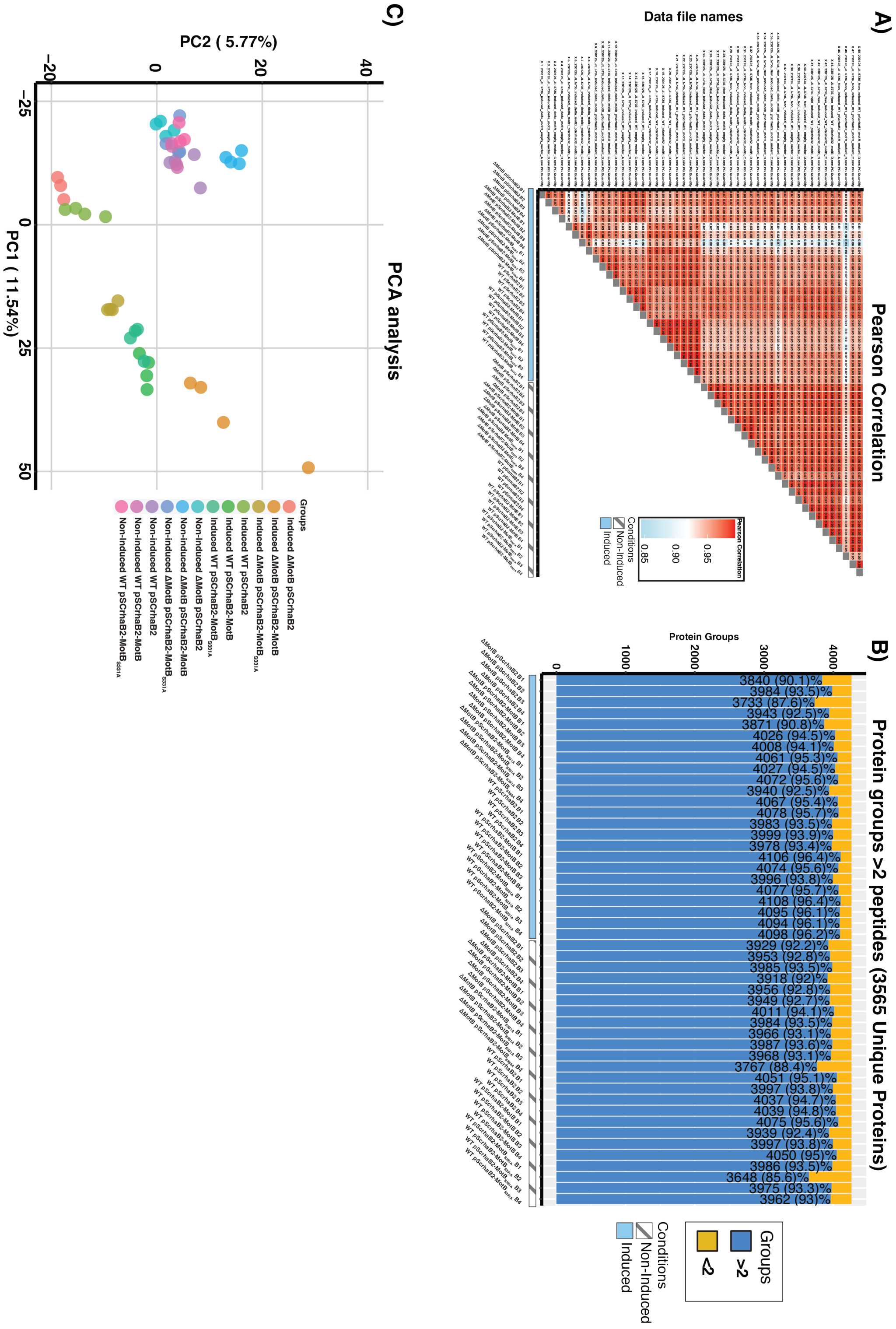

Supplementary Figure 22. QC plots of DIA analysis of *B. cenocepacia* strains containing *motB* complementation vectors and controls. A) Pearson correlation analysis reveals samples have high correlation (>0.90) within individual growth conditions. **B)** A total of 3565 proteins are observed with >90% of these proteins identified with multiple precursors within each replicate. **C)** PCA analysis reveals each biological condition clusters with its corresponding group.

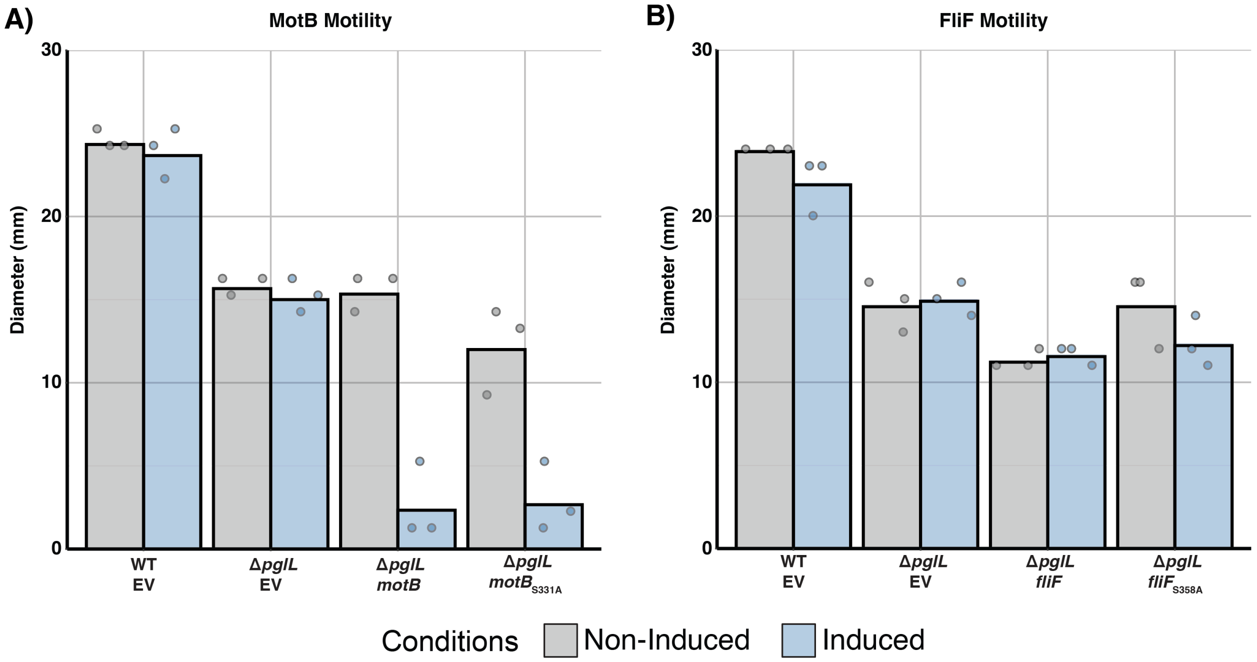

Supplementary Figure 23. Overexpression of *motB* or fliF from pSCrhaB2 does not restore motility in *B. cenocepacia* K56-2 Δ*pglL.* **A)** Motility assays demonstrate the induction of *motB* with rhamnose leads to a reduction in motility within *B. cenocepacia* K56-2 Δ*pglL*. **B)** Motility assays demonstrate the induction of *fliF* expression with rhamnose has no impact on motility within *B. cenocepacia* K56-2 Δ*pglL*.

Westerns Data- Figure 6C & D

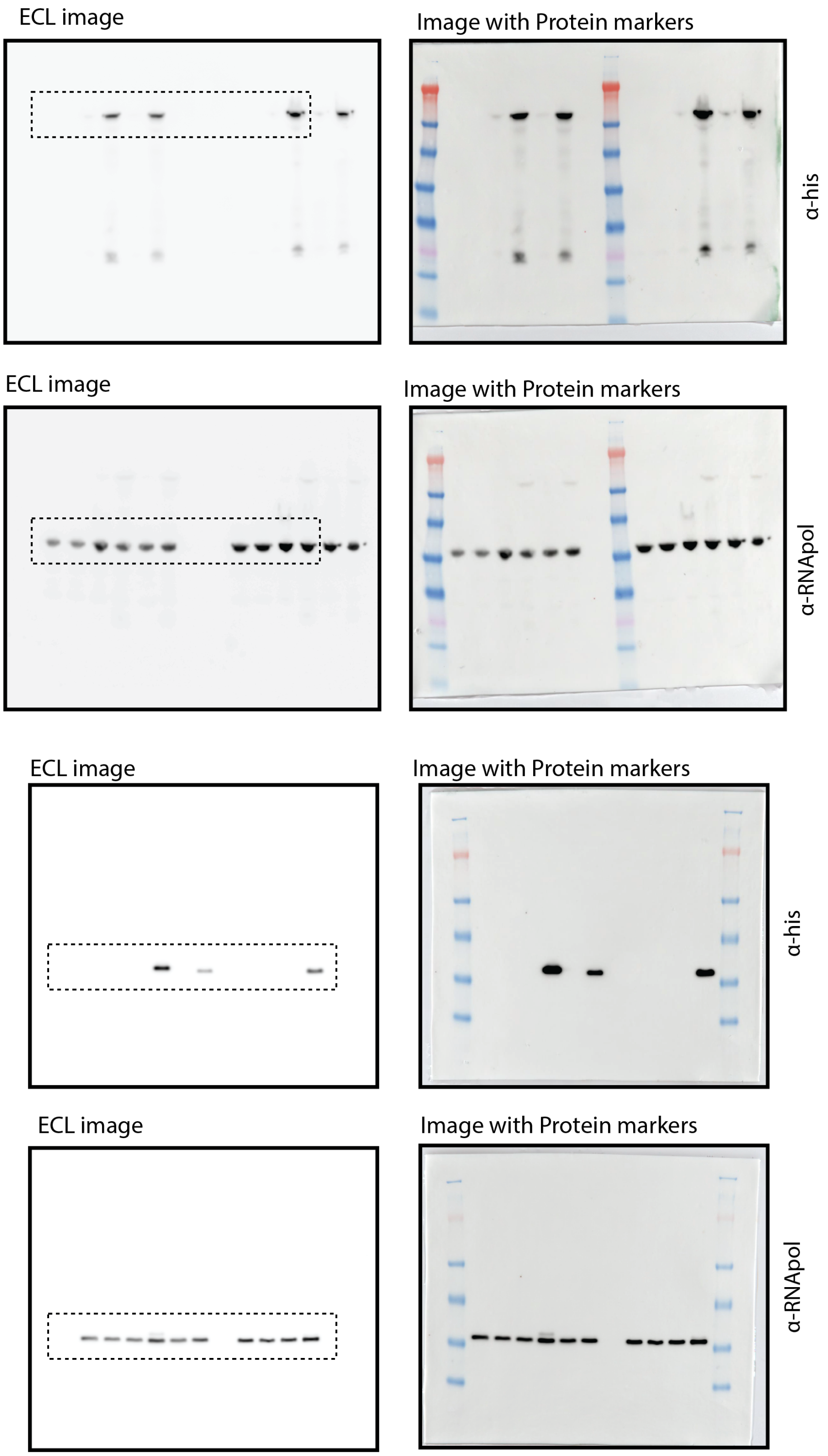

Supplementary Figure 24. Uncropped Western blotting images from Figure 6C and D. The uncropped ECL and membrane images for the western within this manuscript are provided.

References

1 Darling, P., Chan, M., Cox, A. D. & Sokol, P. A. Siderophore production by cystic fibrosis isolates of Burkholderia cepacia. *Infection and immunity* **66**, 874-877 (1998).

2 Oppy, C. C. *et al.* Loss of O-linked protein glycosylation in Burkholderia cenocepacia impairs biofilm formation, siderophore activity and alters transcriptional regulators. *mSphere* **(Accepted)** (2019).

3 Figurski, D. H. & Helinski, D. R. Replication of an origin-containing derivative of plasmid RK2 dependent on a plasmid function provided in trans. *Proceedings of the National Academy of Sciences of the United States of America* **76**, 1648-1652 (1979). https://doi.org:10.1073/pnas.76.4.1648

4 Flannagan, R. S., Linn, T. & Valvano, M. A. A system for the construction of targeted unmarked gene deletions in the genus Burkholderia. *Environ Microbiol* **10**, 1652-1660 (2008). https://doi.org:10.1111/j.1462-2920.2008.01576.x

5 Hamad, M. A., Di Lorenzo, F., Molinaro, A. & Valvano, M. A. Aminoarabinose is essential for lipopolysaccharide export and intrinsic antimicrobial peptide resistance in Burkholderia cenocepacia(dagger). *Molecular microbiology* **85**, 962-974 (2012). https://doi.org:10.1111/j.1365-2958.2012.08154.x

6 Cardona, S. T. & Valvano, M. A. An expression vector containing a rhamnose-inducible promoter provides tightly regulated gene expression in Burkholderia cenocepacia. *Plasmid* **54**, 219-228 (2005). https://doi.org:10.1016/j.plasmid.2005.03.004
